# Towards triptycene functionalization and triptycene-linked porphyrin arrays

**DOI:** 10.3762/bjoc.16.70

**Published:** 2020-04-17

**Authors:** Gemma M Locke, Keith J Flanagan, Mathias O Senge

**Affiliations:** 1 School of Chemistry, SFI Tetrapyrrole Laboratory, Trinity Biomedical Sciences Institute, 152–160 Pearse Street, Trinity College Dublin, The University of Dublin, Dublin 2, Ireland

**Keywords:** BODIPY, Pd-catalyzed cross-coupling, porphyrins, Sonogashira cross-coupling, triptycene

## Abstract

Herein, 9,10-diethynyltriptycene is investigated for its use as a rigid isolating unit in the synthesis of multichromophoric arrays. Sonogashira cross-coupling conditions are utilized to attach various porphyrins and boron dipyrromethenes (BODIPYs) to the triptycene scaffold. While there are previous examples of triptycene porphyrin complexes, this work reports the first example of a linearly connected porphyrin dimer, linked through the bridgehead carbons of triptycene. Symmetric and unsymmetric examples of these complexes are demonstrated and single crystal X-ray analysis of an unsymmetrically substituted porphyrin dimer highlights the evident linearity in these systems. Moreover, initial UV–vis and fluorescence studies show the promise of triptycene as a linker for electron transfer studies, showcasing its isolating nature.

## Introduction

New rigid multiporphyrin architectures are still a key interest to the synthetic chemist due to their potential applications as organic conducting materials, nonlinear optical materials, molecular wires and near-infrared (NIR) absorbers [1–4]. In order to access these multiporphyrin arrays, the porphyrin units have been connected in a number of different ways; from phenylene, ethynyl, ethenyl or alkane linkers [5–8], to meso–β- [9] or β–β-linkages [10] or alternatively by connecting two or more meso–meso-linked porphyrin units via oxidative fusing reactions [11–15]. However, several limitations were encountered such as synthetic inaccessibility, poor solubility, and conformational heterogeneity affecting the feasibility of these constructed porphyrin arrays. In addition, an orthogonal arrangement of the porphyrin units is apparent for porphyrin arrays that are meso–meso linked which, theoretically, can cause a significant energy/charge sink. Moreover, the use of π-conjugated linkers in porphyrin arrays results in significantly altered UV–vis spectra, indicating very strong electronic coupling that causes the loss of individual unit characteristics through delocalization of π-electrons. Consequently, it is necessary to design molecules, capable of achieving an energy- and/or electron-transfer process without causing serious electronic delocalization and/or an energy sink.

A key goal of recent research into multiporphyrin arrays is modulating the absorption profile and electrochemical properties of the porphyrins by increasing the size of the aromatic π-system [16]. In contrast, our approach described herein deviates significantly from previous studies by examining how isolating the individual porphyrin units affects the overall photophysical properties of the system, while maintaining its structural integrity and rigidity. It thus complements the existing research by addressing an area of multiporphyrinoid chemistry that has been neglected thus far.

Used comparatively to the porphyrin molecule, BODIPYs are another class of pyrrolic compounds that are essentially half a porphyrin molecule. These highly fluorescent organic compounds are a part of the difluoroboraindacene family. Owing to their versatility, they have become increasingly popular for their use as fluorophores [17–19]. BODIPY-based dyes have found application in many areas including biological labelling and are known photostable substitutes for fluorescein, giving them applications in cell imaging [20]. Due to the conjugated π-electron system present in BODIPYs they are more electron rich than porphyrins and possess an intense UV–vis absorption at approximately 400 nm, making them ideal candidates to study the potential electron donor–acceptor dyads [19].

**Figure 1:**
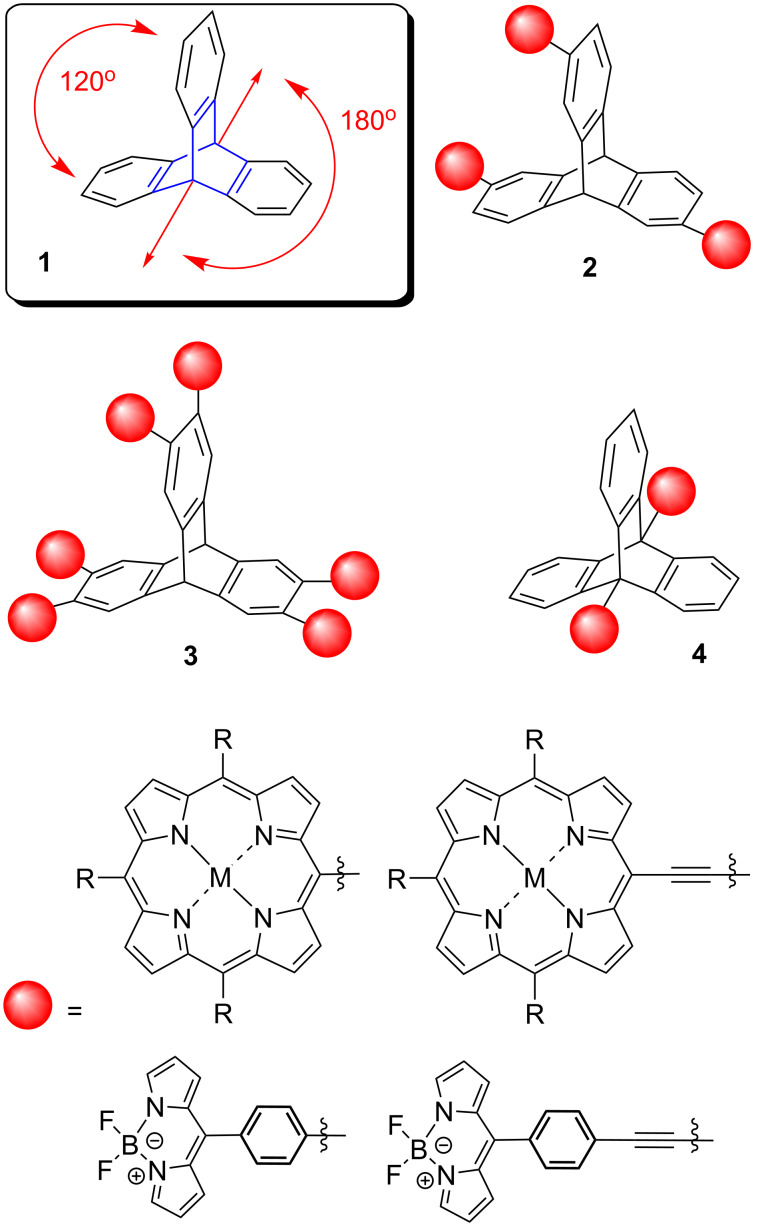
Triptycene as a scaffold and selected porphyrin and BODIPY arrays.

Triptycene (**1**) consists of three benzene rings fused to a bicyclo[2.2.2]octane (BCO) skeleton, it is rigid, isolating and amenable to a wide range of chemical transformations. Due to the 120° rigid void, mono-, di-, tri-, tetra-, penta- and hexa-functionalizations of triptycene scaffolds can be achieved in a spatially defined manner (Figure 1) [21–23]. Thus, a variety of functional groups can be presented in fixed orientations [24]. The periphery of triptycene was successfully functionalized when multidye triptycene-linked complexes such as **2** were synthesized by us with the purpose of conducting electron transfer studies [25]. Both Suzuki and Sonogashira cross-coupling reactions were employed to realize this new class of triptycene-linked trimeric porphyrins. The three porphyrins, or three BODIPYs in **2** were either linked directly to the periphery of triptycene or via various linkers; arrays with six chromophores **3** have thus far eluded us [26–27].

While much work has been done on the functionalization of the aromatic rings of triptycene as in **2** or **3** [24–30], the attachment of chromophores at the bridgehead positions has yet to be realized. Functionalization of the bridgehead positions of triptycene (as in **4**) allows for the attachment of moieties in a 180° linear fashion, subsequently accessing the molecular rotation observed in several other linearly substituted triptycenes [31]. Attaching porphyrins to the bridgehead positions in a linear fashion not only allows for compounds with well-defined spatial arrangements but additionally, the non-conjugated character of the triptycene sp^3^-carbon atoms prevents through-bond electronic communication and undesirable orbital overlap [32–33]. Therefore, linearly linked triptycene porphyrin complexes could lead to compounds with the required conformation, geometry and spatial arrangement necessary for use in electron transfer studies. For further information on other bridgehead modifications of the triptycene scaffold see the comprehensive review on this topic [33].

## Results and Discussion

### Synthesis of triptycene precursors

Initially, to synthesize linearly linked triptycene porphyrin dimers, the attachment of various linker groups at the triptycene bridgehead carbon atoms was investigated. The initial strategy was to functionalize 9,10-dibromotriptycene [34] directly through various Pd-catalyzed or organocopper cross-coupling [35] reactions. However, exploratory studies did not show much promise and this approach was abandoned, as was the functionalization of 9,10-dibromoanthracene with phenyl spacers prior to triptycene formation (see Supporting Information File 1 for full experimental data). Instead we focused on the synthesis of systems with ethynyl spacers between the triptycene bridgehead positions and chromophores. To allow for the generation of symmetric and unsymmetric dyads 9,10-diethynyltriptycene **5** and its anthracene precursor were synthesized as per a literature procedure [36] while, 9-[(triisopropylsilyl)ethynyl]-10-ethynyltriptycene (**12**) and its precursors were synthesized according to the procedure from Toyota et al. [37], respectively.

#### Synthesis of triptycene-linked porphyrin dimers

**Scheme 1:**
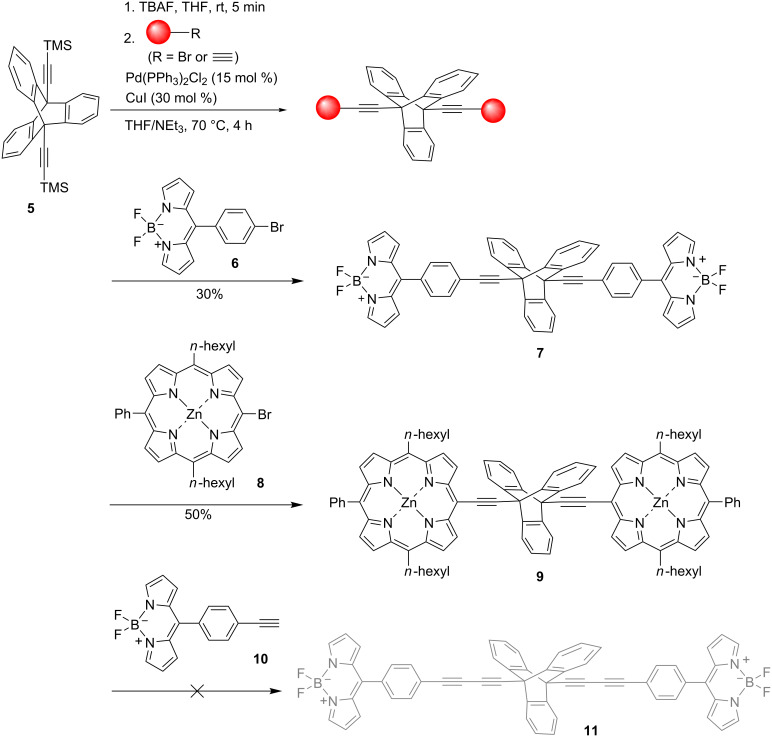
Sonogashira cross-coupling reactions to form symmetric porphyrin and BODIPY triptycene-linked dyads.

**Scheme 2:**
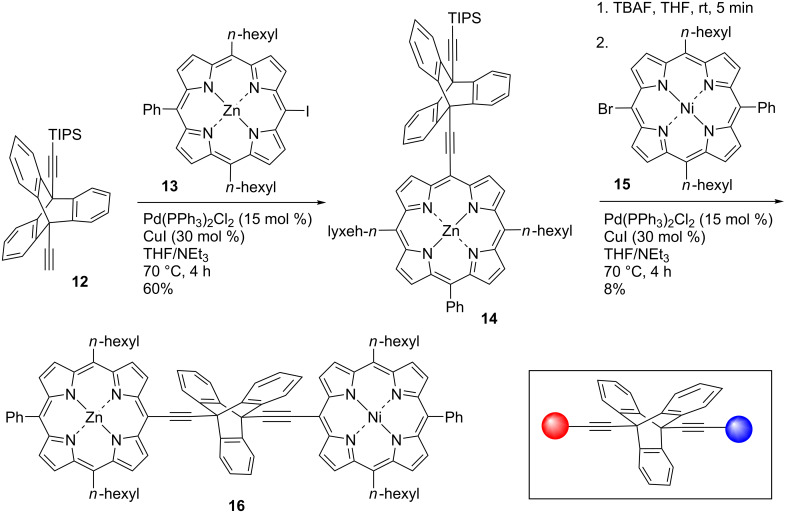
Sonogashira cross-coupling reactions to form a triptycene-substituted porphyrin monomer and an unsymmetrically substituted triptycene-linked porphyrin dyad.

With the introduction of synthetic handles to the bridgehead positions of triptycenes in **5** (Scheme 1) and **12** (Scheme 2), efforts were made to attach various chromophores in order to investigate the effects of triptycene as a linker for electron transfer properties. With the goal of synthesizing various new triptycene-linked porphyrin and BODIPY dimers, ethynyltriptycene **5** was deprotected with TBAF (1 M) to allow for coupling with the relevant porphyrin/BODIPY. Standard Sonogashira cross-coupling conditions were followed with the catalyst Pd(PPh_3_)_2_Cl_2_, co-catalyst CuI and NEt_3_ as a base. Once TLC analysis indicated the completion of the reaction, the crude material was purified via column chromatography using silica gel. The BODIPY dimer **7** was prepared using **6** [38] and was obtained as a fluorescent orange solution or as pink-orange crystals in a 30% yield. Similarly, the Pd-catalyzed Sonogashira cross-coupling reaction [39] was successful for the reaction with porphyrin **8** [40], resulting in a yield of 50% of the purple-green product **9**. An additional reaction was carried out via a Glaser coupling with BODIPY **10** in order to extend the distance between the two BODIPY chromophores but the desired product **11** could not be detected by spectroscopic techniques.

In an effort to synthesize an unsymmetrically substituted porphyrin dimer, triptycene **12** was utilized (Scheme 2). Firstly, the zinc porphyrin **13** [41] was coupled to triptycene **12** using Sonogashira methods that were previously successfully employed with compound **11**. The desired compound **14** could be isolated as a purple solid in 60% yield. Subsequently, an in situ deprotection of the TIPS group was carried out with a solution of the triptycene-linked porphyrin **14**. The polarity of the in situ deprotected product and TIPS protected starting material were very similar so TLC analysis was unable to confirm full conversion. For this reason, it may be that the ethynyl bond was not completely deprotected before the next coupling reaction. After the TBAF deprotection step, the second Sonogashira cross-coupling reaction was carried out with the nickel porphyrin **15** [41] under the same reaction conditions as before. After silica gel column chromatography, a dark purple solid of **16** was isolated in 8% yield.

**Scheme 3:**
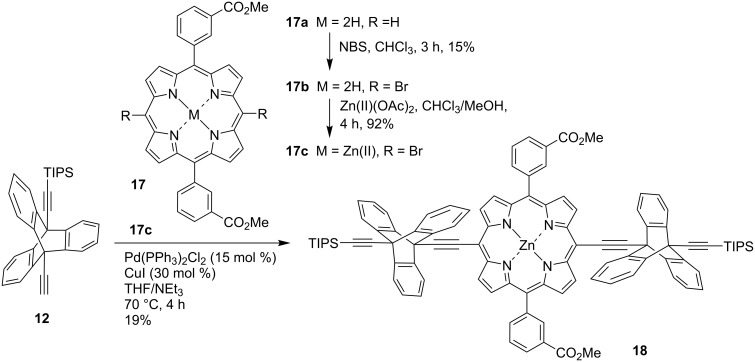
Synthesis of the triptycene-porphyrin-triptycene complex **18**.

The unsymmetrically substituted triptycene **12** was utilized for several other cross-coupling reactions, including one with the dibrominated porphyrin **17c** which was prepared via metalation of **17b** and bromination of **17a** [42] (Scheme 3). The coupling reaction was initially carried out with (5,15-dibromo-10,20-dihexyl/diphenyl)porphyrinato)zinc(II) but both porphyrins had extremely low solubility. Using the more soluble **17c** gave access to the triptycene–porphyrin–triptycene complex **18**. The reaction was carried out using standard Sonogashira cross-coupling conditions and the product was isolated as a green solid in 19% yield. A major side-product of the reaction was the Glaser product, i.e., dimerization of two ethynyl triptycene molecules **12**. The synthesis of this molecule offers future avenues to extend this family of triptycene-linked porphyrins from dimers to trimers.

#### Single crystal X-ray analyses

**Figure 2:**
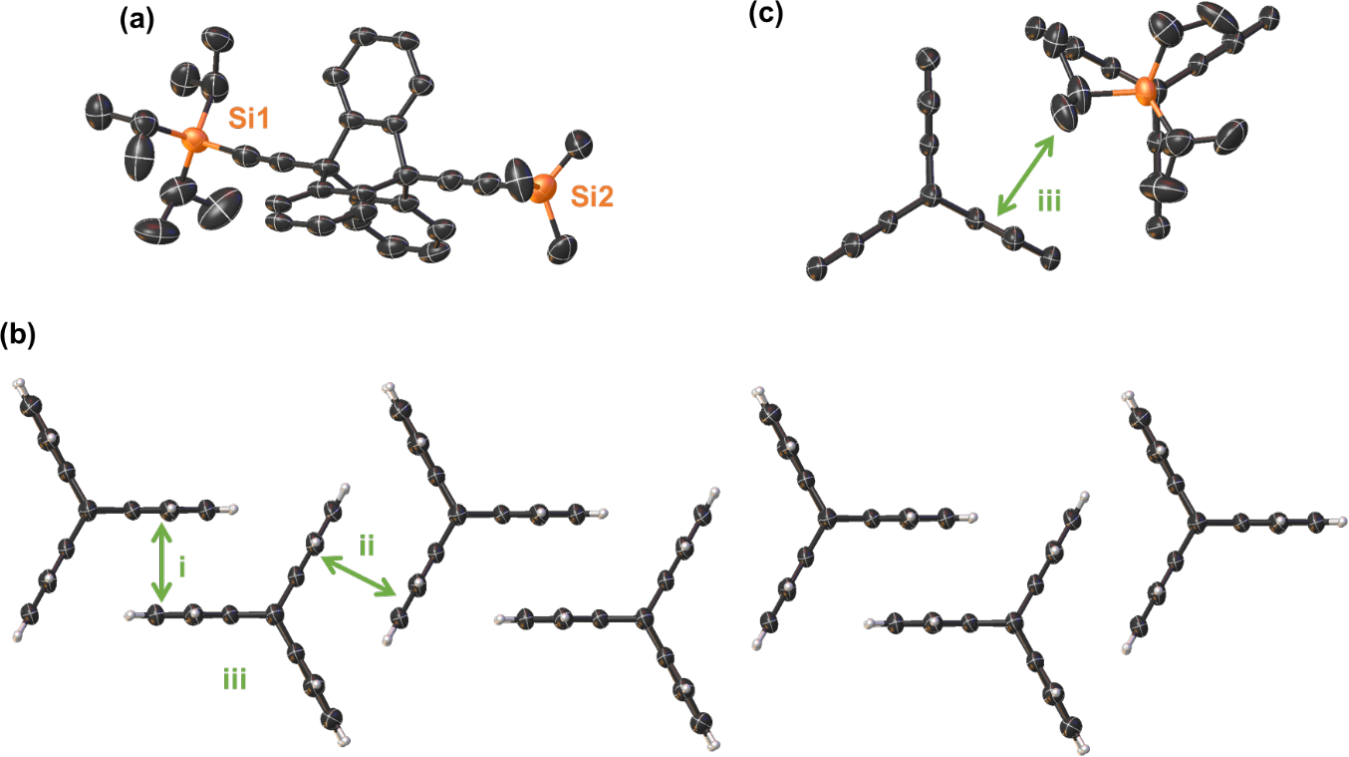
Single crystal X-ray structure of triptycene **5**. (a) Molecular structure of **5** in the crystal with hydrogen atoms and minor disorder omitted for clarity (thermal displacement 50%); (b) expanded view showing the π-stacking between the i and ii rings (thermal displacement 50%). Bridgehead substituents have been omitted for clarity; (c) expanded view showing C–H···π interactions between the TIPS group of one molecule and the third pocket (iii) of another triptycene molecule. Hydrogen atoms and minor disorder omitted for clarity (thermal displacement 50%).

To further confirm the structures of the substituted triptycene, we obtained single crystals suitable for analysis of the disubstituted triptycenes **5** and **16**. The structure of **5** was solved with one molecule in the asymmetric unit with the bridgehead substituents being almost 180° to each other (Figure 2, for labelling see Figure S33 in Supporting Information File 1). Unlike the unsubstituted triptycene **1** crystal structure which exhibits a high degree of C–H···π interactions between the aromatic rings [43], there is a high degree of interdigitation between the aromatic rings of the triptycene unit (Figure 2b) akin to some triptycene-quinones [44]. This is due to two of the triptycene aromatic rings i [C8A to C8, centroid···centroid distance of 3.845(3) Å and shift distance of 1.507(5) Å] and ii [C11 to C16, centroid···centroid distance of 3.755(3) Å and shift distance of 1.593(5) Å] involved in a head-to-head slip plane π-stacking interaction with another triptycene molecule either side, while the third face ring iii of the triptycene exhibits C23–H23B···π interactions between the isopropyl group of the TIPS at distances of 2.860(1) Å effectively blocking this location from interacting in a similar fashion (Figure 2c). This pattern is also reflected in the crystal packing (Figure S34 in Supporting Information File 1). This motif is similar to other previously reported bridgehead-substituted triptycene structures such as 9-iodotriptycene. However, this design is limited as with larger functional groups at the bridgehead position impose alternate packing patterns [45].

**Figure 3:**
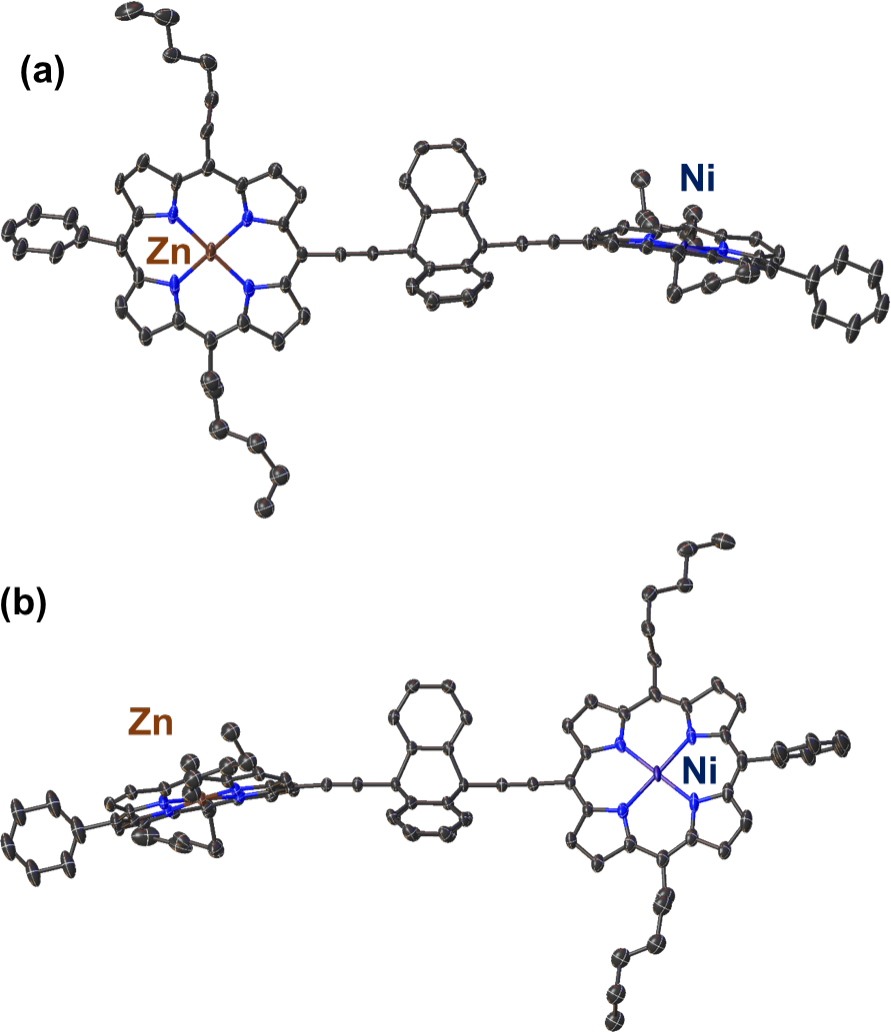
Single crystal X-ray structure of triptycene-linked zinc-nickel porphyrin dimer **16** showing the conformation within the crystal structure; (a) top-view of zinc porphyrin; (b) top-view of nickel porphyrin. Hydrogen atoms and minor disorder are omitted for clarity (thermal displacement 50%).

The structure of the porphyrin–triptycene–porphyrin dimer **16** (Figure 3) was solved with one molecule in the asymmetric unit (for labelling see Figure S35 in Supporting Information File 1). In this structure, the two porphyrin residues are held at 74.1(1)° rotation to one another and at a meso–meso distance of 10.823(6) Å, while the bridgehead substituents are held in a linear fashion (≈180°) around the triptycene molecule.

**Figure 4:**
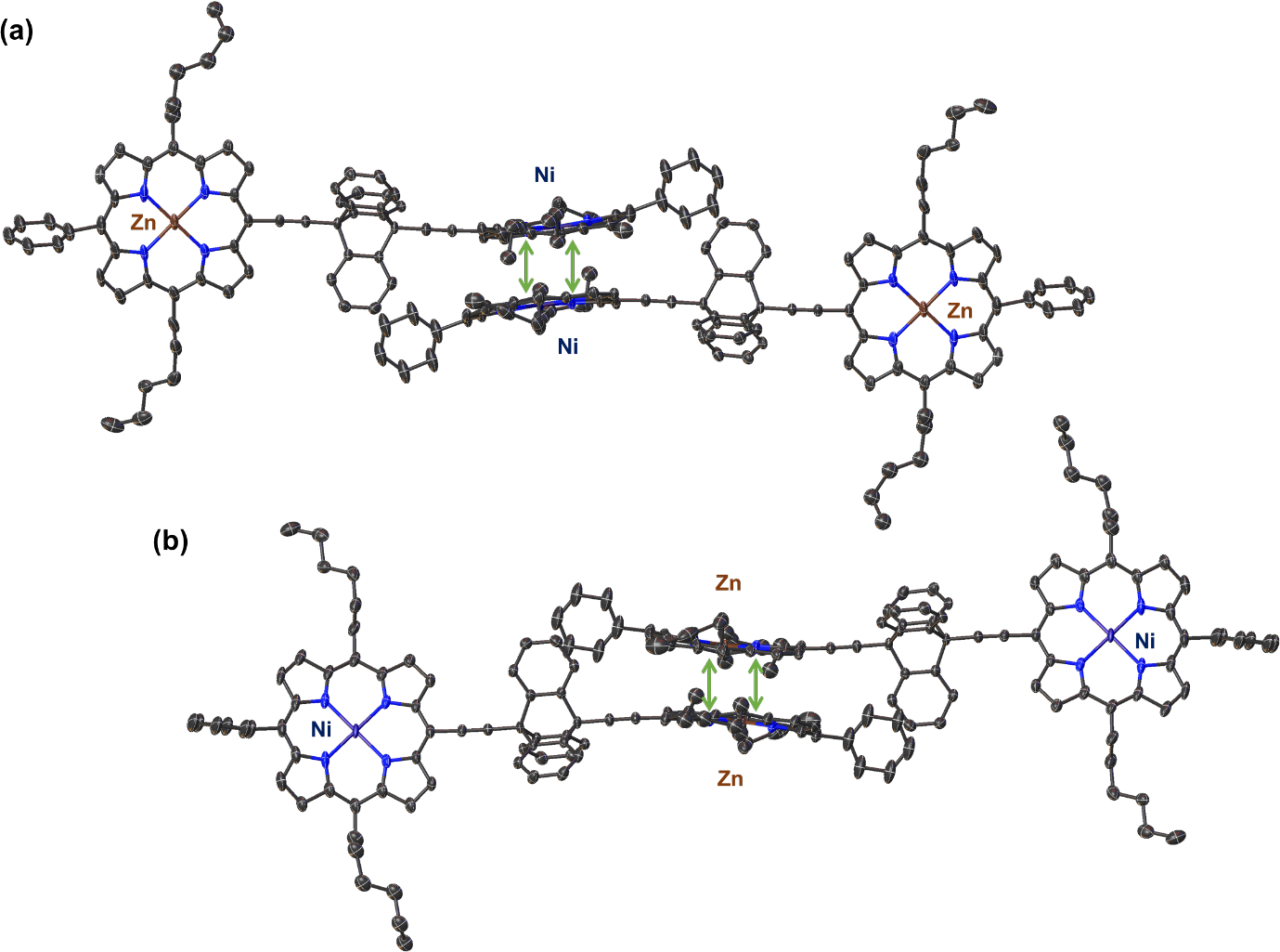
Views of the single crystal X-ray structure of triptycene-linked zinc-nickel porphyrin dimer **16** showing the π-stacking homo-metallic interactions between Ni(II)-porphyrin···Ni(II)-porphyrin (a) and Zn(II)-porphyrin···Zn(II)-porphyrin (b). Hydrogen atoms and minor disorder are omitted for clarity (thermal displacement 50%).

**Figure 5:**
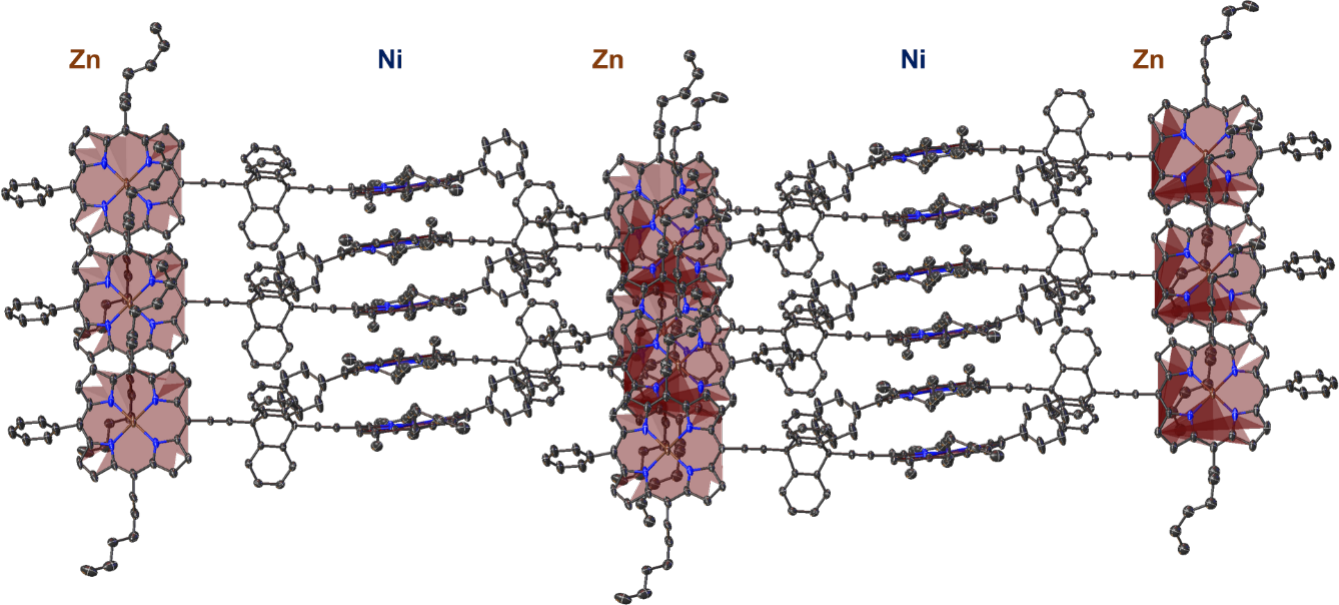
Expanded structure view of the triptycene-linked zinc-nickel porphyrin dimer **16** showing the repeating Zn···Ni···Zn···Ni network in the crystal lattice. Hydrogen atoms and minor disorder are omitted for clarity (thermal displacement 50%). Porphyrin macrocycle mean-planes are indicated by maroon shading.

In this structure, it should be noted that there is a high degree of π-stacking between the porphyrin macrocycle. This is highlighted in Figure 4; however, there is a clear preference for the homo-metallic selective interactions between the porphyrin rings. As such, in the crystal packing the Ni(II)-porphyrin only π-stacks with another Ni(II)-porphyrin at a centroid to mean-plane distance of 3.421(5) Å and a shift of distance of 3.415(8) Å (Figure 4a). The Zn(II)-porphyrin only π-stacks with another Zn(II)-porphyrin at a centroid to mean-plane distance of 3.399(2) Å and a shift distance of 3.378(6) Å (Figure 4b). This is further exemplified in the expanded structure view in Figure 5 where it can be clearly seen that the porphyrin rings are aligned in a Zn···Ni···Zn···Ni type pattern throughout the crystal lattice. This pattern is also clearly visible in the crystal packing (Figure S36 in Supporting Information File 1). While this feature is an interesting take on the selective homo interactions, the explanation of this motif formation is potentially more mundane in this case. Considering that during the crystallization process, the spontaneous formation of the homo or hetero π-stacking of the porphyrin rings are both possible. However, due to the sever 74.1(7)° rotation between the two 24-atom mean planes of the porphyrin macrocycles and the space occupied by the triptycene, creating a hetero π-stacking would form larger voids in the crystals structure. This would be less favored than the tight packing observed for the homo π-stacking. Similarly, a mixture between the homo and hetero π-stacking would cause a more amorphous crystal and would be the least likely to form in a solid state a crystal suitable for determination by X-ray diffraction. The driving force for this crystal packing is the π-stacking between the porphyrin rings and not the interactions between metal ions. An additional feature in this structure is the interactions between the aromatic triptycene rings and the phenyl rings of the C15-meso-position of the porphyrin (see Figure S37 in Supporting Information File 1). This is seen between the associative interactions (a) and (b) with a centroid-to-plane distance 2.031(9) Å and a shift distance of 4.294(5) Å and interactions (c) and (d) with a centroid-to-plane distance 3.418(11) Å and a shift distance of 2.446(14) Å.

#### Absorption and steady-state emission properties of triptycene complexes

**Figure 6:**
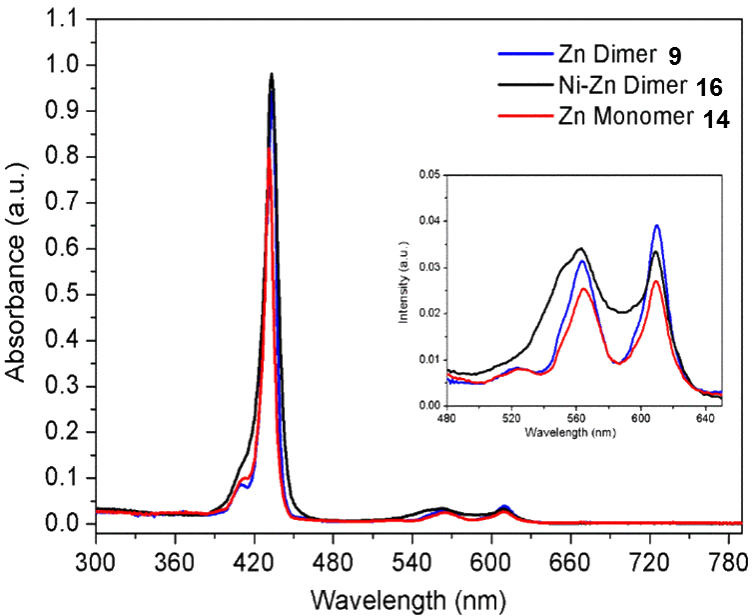
UV–vis of symmetric and unsymmetric triptycene porphyrin dimers **9** and **16**, and the triptycene porphyrin monomer **14** in CHCl_3_.

The photophysical properties of the five new triptycene-liked porphyrin/BODIPY compounds **7**, **9**, **14**, **16** and **18** were investigated in preliminary studies for their suitability as model compounds for electron transfer in the context of artificial photosynthesis. Similar Soret band positions were seen for the symmetric zinc dimer **9**, the unsymmetrical dimer **16** and the zinc monomer **14** at 431 (23202), 432 (23149) and 433 (23095) nm (cm^−1^) in CHCl_3_, respectively (Figure 6). A similar Soret band for both the dimer and monomer **9** and **14** suggested that the two subunits in the dimer were not connected through efficient electronic conjugation/delocalization. Two Q bands were observed in all cases, which is characteristic of zinc porphyrins due to the increased symmetry in the compound as compared to the free base derivatives, which ordinarily feature four Q bands [46]. While the Q bands observed for the zinc dimer and monomer had low full-width at half-maxima (FWHM) of ≈26 nm (3.85 × 10^5^ cm^−1^), i.e., a narrow band, the zinc-nickel dimer **16** exhibited a high FWHM of ≈50 nm (2 × 10^5^ cm^−1^), potentially caused by overlapping of the nickel and zinc porphyrin Q bands which could be due to the modulation of frontier molecular orbitals. The difference in the FWHM of the Q bands of the symmetric and unsymmetric dimers, **9** and **16**, respectively, is nearly double, which is a significant difference in comparison to the Soret bands that exhibit FWHM of 10 nm (1 × 10^6^ cm^−1^) for the two dimers and 8 nm (1.25 × 10^6^ cm^−1^) for the monomer.

**Figure 7:**
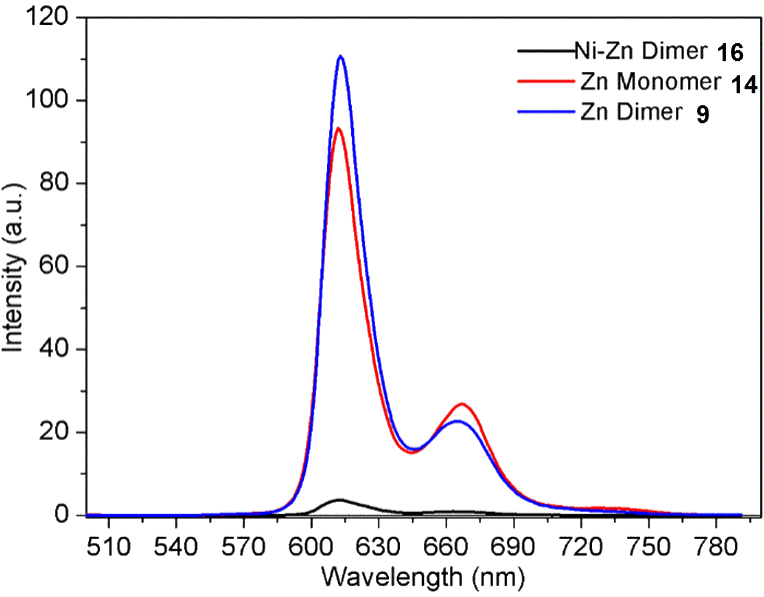
Emission spectrum of symmetric and unsymmetric triptycene-linked porphyrin dimers **9** and **16**, and a triptycene-linked porphyrin monomer **14** in CHCl_3_ (all compounds were excited at λ = 430 nm).

The difference in properties of the unsymmetric zinc-nickel dimer **16** is observed significantly more in the corresponding fluorescence emission spectrum (Figure 7). The emission observed for the zinc monomer and dimer, **14** and **9**, respectively, was significantly more enhanced, in comparison to the practically quenched fluorescence detected for the zinc-nickel dimer **16** which may be due to the electron-deficient nature of the nickel porphyrin [47]. In this instance, the excited zinc porphyrin is acting as electron donor while the electronically inactive nickel porphyrin is acting as the acceptor. An electron/energy transfer is occurring between the two porphyrins, therefore, when the molecule is excited at the wavelength of the zinc porphyrin, the fluorescence emission ordinarily observed for the zinc porphyrin does not occur as the energy has been transferred to the nickel porphyrin, which subsequently quenches the fluorescence due to the heavy atom effect that is characteristic of nickel [48]. These initial results suggest that an energy/electron transfer is taking place, as the strong emission of the excited zinc porphyrin that was observed for the zinc monomer and dimer, **14** and **9**, respectively, does not occur in the unsymmetric system. Due to the differently substituted *meso*-positions present on all the porphyrins for the three compounds **9**, **14** and **16**, the emission spectra observed have signals that are unequal in intensity, which is characteristic of unsymmetric porphyrin units. In contrast, the emission spectra seen for a symmetric porphyrin like 5,10,15,20-tetraphenylporphyrin has two signals of equal intensity [49]. As previously mentioned, the zinc monomer and dimer, **14** and **9**, show similar fluorescence emissions. This is due to the presence of the non-conjugated triptycene linker in the dimer **9**, causing the two porphyrins to be electronically isolated and akin in environment to the porphyrin of the zinc monomer **14**.

**Figure 8:**
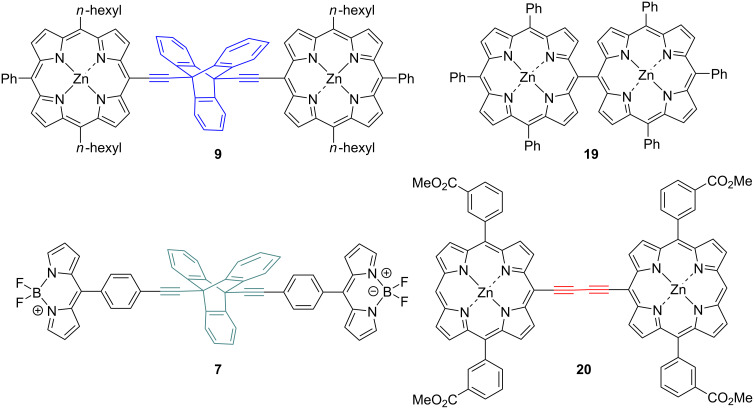
Compounds used for spectroscopic comparisons.

**Figure 9:**
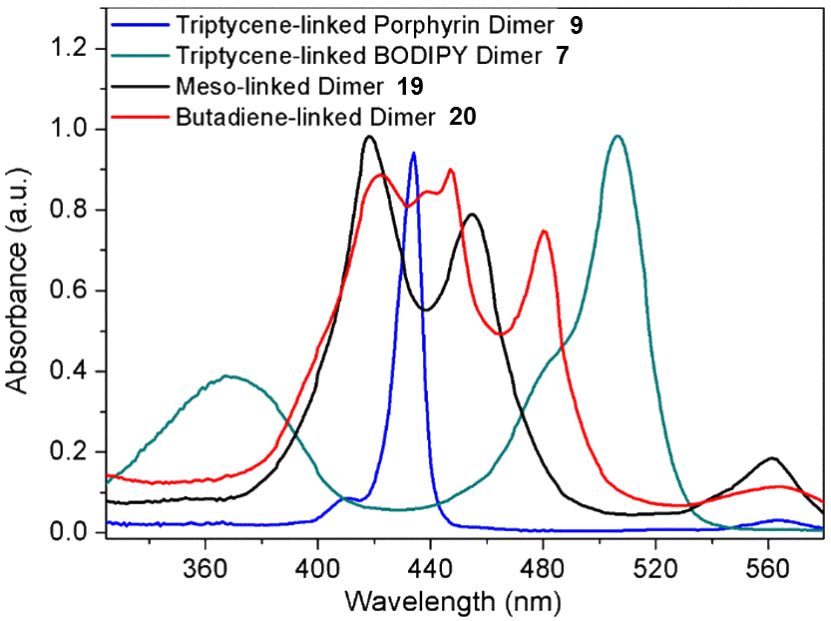
UV–vis spectra of various porphyrin/BODIPY dimers with different linker groups in CHCl_3_.

**Figure 10:**
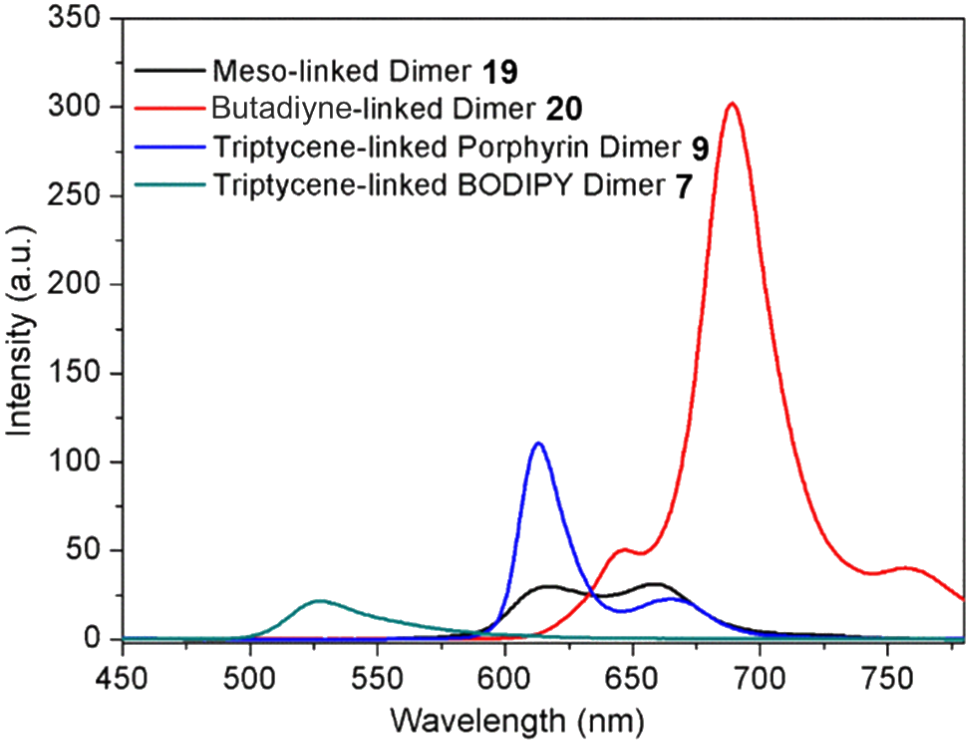
Fluorescence emission spectrum of various porphyrin/BODIPY dimers with different linker groups in CHCl_3_. The excitation for compounds **9**, **7**, **19** and **20** was λ = 433 (23095), 370 (27027), 455 (21978) and 450 (22222) nm (cm^−1^), respectively.

To effectively demonstrate the significance and purpose of utilizing triptycene as a rigid linear linker, the UV–vis and fluorescence emission spectra of related dimers (Figure 8), connected via different linker groups, were taken (Figure 9 and Figure 10). Both the triptycene-linked zinc porphyrin dimer **9** and BODIPY dimer **7** were compared with a meso–meso-linked dimer **19** [50] and butadiyne-linked dimer **20** [51]. The UV–vis spectra seen in Figure 9 highlight the diversity in composition of the four compounds under investigation. The triptycene-linked zinc dimer **9**, previously discussed, has a Soret band at 433 nm (23095 cm^−1^) and two Q bands at 564 nm (17731 cm^−1^) and 610 nm (16393 cm^−1^), respectively, while the triptycene-linked BODIPY dimer **7** has a band at 367 nm (27248 cm^−1^) and another more intense band at 506 nm (19763 cm^−1^). In both these instances, the compounds show clear and distinct signals due to the absence of any electronic delocalization. The BODIPY unit was selected as a means for comparison with the triptycene-linked porphyrin dimers as they exhibit high fluorescence emissions.

Owing to the orthogonal orientation of the two porphyrins, distinct signals were also observed for the meso–meso-linked dimer **19**. The effects of an orthogonal arrangement of the two subunits meso–meso-linked porphyrin dimers is well-known in the literature [4] and results in two distinct Soret bands seen at 418 (23923) and 455 (21978) nm (cm^−1^) as each porphyrin is electronically isolated due to the lack of orbital overlap between the two units. In stark contrast, the linearly linked butadiyne dimer **20** maintains conjugation between the two porphyrin units resulting in very strong electronic coupling, i.e., losing the characteristic of individual units due to delocalization of π-electrons. The Soret bands of the four different conformations of the porphyrins in the butadiyne-linked dimer **20** are barely distinguishable from one another at 422 (23697), 439 (22779), 447 (22371) and 480 (20833) nm (cm^−1^), due to the electronic coupling present in the system. This effect is even more notable for the dimer’s Q bands which cannot all be distinguished.

Similar differences between these four compounds are seen in the fluorescence emission spectra (Figure 10). The most notable distinction between the compounds is in the emission values observed. The BODIPY dimer **7** shows the lowest emission of the four compounds with only one emission band seen at 527 nm (18975 cm^−1^). The symmetric meso–meso-linked dimer **19** exhibits a similar emission at 617 nm (16207 cm^−1^) with two equally intense bands (a characteristic of symmetric porphyrins). The nearly similar λ_max_ of both these dimers reinforces the lack of extended π-conjugation or communication that is present in these molecules. Comparatively, the zinc dimer **9** has an increased emission seen at 617 nm (16207 cm^−1^), while the butadiyne-linked dimer **20** distinguishes itself again with a notably enhanced emission at 688 nm (14535 cm^−1^). This is again due to the orbital overlap between the two moieties resulting in an enhanced emission. The λ_max_ of butadiyne-linked dimer **20** is red-shifted 71 (1.4 × 10^5^) and 75 (1.3 × 10^5^) nm (cm^−1^) with respect to the zinc porphyrin dimer **9** and the meso–meso-linked dimer **19**. The difference in emission is potentially due to the combined effect of the four electron-withdrawing methyl ester groups of the porphyrins of **20**, but more likely due to the extended conjugation present between the two linearly linked porphyrin units. Extended conjugation causes increased planarity and conjugation in the system, along with a reduction in the HOMO–LUMO gap resulting in the red-shifted spectra. In contrast to the porphyrin emission spectra, BODIPYs exhibit a blue-shifted spectrum with lower Stokes Shift values, enabling a different frequency of light to be accessed. Overall, there is a significant difference in the HOMO–LUMO gap of the three porphyrin dimers. The meso–meso-linked dimer **19** has the largest gap of 2.21 eV, followed by the triptycene-linked dimer **9** with a gap of 2.03 eV and the butadiyne-linked dimer **20** with 1.87 eV. These values give an initial indication of the extent of π-conjugation occurring within these systems, as the more conjugation present in the system the lower the HOMO–LUMO gap. A spectroscopic comparison of the porphyrin-triptycene-porphyrin dimer **7** and the triptycene-porphyrin-triptycene dimer **18** is given in Supporting Information File 1.

## Conclusion

After multiple attempts to synthesize a functionalized 9,10-disubstituted-triptycene, 9,10-diethynyltriptycene was synthesized, enabling access to a series of symmetric porphyrin/BODIPY dimers. Moreover, the synthesis of one unsymmetric porphyrin dimer **16** and a porphyrin-linked bistriptycene **18** was also achieved. A single crystal X-ray structure of the former highlighted the evident linearity in the system and arrangement of the porphyrins at a dihedral angle of 66.84° with respect to one another. Various π–π stacking interactions were also observed between the zinc porphyrins, the nickel porphyrins and a triptycene face with a porphyrin phenyl ring. UV–vis and fluorescence studies of five of the synthesized compounds were conducted and showcased the isolating properties of the triptycene scaffold, made apparent when compared to other linker groups such as in the butadiyne- and meso–meso-linked complexes **20** and **19**, respectively, where either the individual characteristic of the porphyrin units were diminished due to delocalization of π-electrons or chemically isolated due to the inherent orthogonality in the system. Preliminary evidence of electron transfer was also observed with the unsymmetric zinc-nickel complex **16** as a drastically reduced emission of the excited zinc porphyrin was observed, when excited at a wavelength corresponding to the zinc porphyrin, owing to electron transfer from the zinc porphyrin unit to the nickel porphyrin unit.

Initial studies have shown the promise of triptycene as a linker for electron transfer studies, and an expanded library could lead to further interesting results where the linearity, hybridization and accessibility of triptycene together with dyads in the bridgehead ‘rotor’ position can be further highlighted. While 9,10-diethynyltriptycene offers an initial steppingstone into obtaining such multichromophoric arrays, further investigation into alternative functional groups, such as boronic esters and aromatic units, to functionalize triptycene need to be conducted to widen the scope of this unit as a viable scaffold.

## Experimental

General experimental conditions and methods are given in the supplementary material (see Supporting Information File 1 for full details).

### General procedure 1: (in situ deprotection with TBAF and Sonogashira cross-coupling of alkynyltriptycene and porphyrins/BODIPY)

TMS-protected triptycene was dissolved in THF in a 25 mL Schlenk tube at room temperature under an inert atmosphere. TBAF (1 M solution) was added dropwise and the reaction stirred for five minutes until TLC analyses indicated complete consumption of starting material. NEt_3_ was added by syringe in a 3:1 ratio of THF to NEt_3_. The solution was bubbled with argon for five to ten minutes before PdCl_2_(PPh_3_)_2_ and CuI were added. The reaction mixture was heated to 70 °C for four hours and then diluted with CH_2_Cl_2_ (10 mL) before removal of solvents in vacuo. The crude material was purified by filtration through a short silica gel plug using CH_2_Cl_2_ as eluent to remove any unreacted starting material.

#### Synthesis of 9,10-bis-[4'-ethynyl(phenyl)-1'-borondipyrromethene]triptycene (**7**)

Synthesized by general procedure 1 from triptycene **5** (23 mg, 50 μmol), 1 M TBAF (150 μL, 150 μmol), BODIPY **6** (43.5 mg, 125 μmol), PdCl_2_(PPh_3_)_2_ (3.4 mg, 4.8 μmol) and CuI (1.7 mg, 8.9 μmol) in THF/NEt_3_ (2 mL:0.66 mL). The reaction was stirred for four hours at 70 °C*.* The crude material was purified by filtration with a silica gel column using CH_2_Cl_2_:*n*-hexane (2:1, v/v). Recrystallization (CHCl_3_/*n*-hexane) gave red crystals (12.5 mg, 30%). Mp >350 °C. *R*_f_ = 0.75 (SiO_2_, CH_2_Cl_2_/*n*-hexane 3:1, v/v); ^1^H NMR (600 MHz, CDCl_3_) δ 8.03–7.99 (m, 8H, α-pyrrole-*H* and phenyl-*H*), 7.88 (dd, *J* = 5.2, 3.0 Hz, 6H, triptycene-*H*), 7.72 (d, 4H, phenyl-*H*), 7.19 (dd, *J* = 5.2, 3.0 Hz, 6H, triptycene-*H*), 7.03 (d, 4H, pyrrole-*H*), 6.62 ppm (s, 4H, pyrrole-*H*); ^13^C NMR (151 MHz, CDCl_3_) δ 146.4, 144.7, 143.5, 135.0, 134.3, 132.3, 131.6, 130.9, 126.3, 126.2, 125.6, 122.5, 119.0, 92.3, 86.4, 29.8; ^11^B NMR (128 MHz, CDCl_3_) δ 0.32 (t, *J* = 28.7 Hz); ^19^F NMR (377 MHz, CDCl_3_) δ −145.04 (dd, *J* = 57.5, 28.7 Hz); IR (neat)/cm^−1^) ν̃: 2921 (w), 2851 (w), 1560 (m), 1535 (m), 1412 (m), 1386 (s), 1257 (s), 1154 (m), 1103 (s), 1070 (s), 982 (m), 912 (m), 750 (s), 739 (m), 638 (m), 580 (w); UV–vis (CHCl_3_): λ_max_ [nm] (log ε): 367 (5.57), 506 (5.97); HRMS–MALDI (*m*/*z*): [M]^+^ calcd for C_54_H_32_N_4_F_4_B_2_, 834.2749; found, 834.2748.

#### Synthesis of 9,10-bis[5-ethynyl(10,20-dihexyl-15-phenylporphyrinato zinc(II))]triptycene (**9**)

Synthesized by general procedure 1 from triptycene **5** (23 mg, 50 μmol), TBAF (150 μL, 150 μmol), bromoporphyrin **8** (87 mg, 125 μmol), PdCl_2_(PPh_3_)_2_ (3.4 mg, 4.8 μmol) and CuI (1.7 mg, 8.9 μmol) in THF/NEt_3_ (2 mL:0.66 mL). The reaction mixture was stirred for four hours at 70 °C*.* The crude material was purified by filtration with a silica gel column using 6:1 *n*-hexane/EtOAc. Recrystallization (CHCl_3_/*n*-hexane) yielded purple crystals (40 mg, 50%). Mp 332–335 °C. *R*_f_ = 0.54 (SiO_2_, CH_2_Cl_2_/*n*-hexane, 2:1, v/v); ^1^H NMR (400 MHz, CDCl_3_) δ 10.11 (d, *J* = 4.5 Hz, 4H, *H*_β_), 9.62 (d, *J* = 4.5 Hz, 4H, *H*_β_), 9.38 (d, *J* = 4.6 Hz, 4H, *H*_β_), 8.79 (d, *J* = 4.6 Hz, 4H, *H*_β_), 8.60 (dd, *J* = 5.5, 3.3 Hz, 6H, triptycene-*H*), 8.12 (d, *J* = 6.2 Hz, 4H, phenyl-*H*), 7.70–7.64 (m, 6H, phenyl-*H*), 7.37 (dd, *J* = 5.5, 3.3 Hz, 6H, triptycene-*H*), 4.98–4.92 (m, 8H, hexyl-C*H*_2_), 2.51–2.48 (m, 8H, hexyl-C*H*_2_), 1.84–1.76 (m, 8H, hexyl-C*H*_2_), 1.52–1.44 (m, 8H, hexyl-C*H*_2_), 1.39–1.30 (m, 8H, hexyl-C*H*_2_), 0.87 ppm (t, *J* = 7.3 Hz, 12H, hexyl-C*H*_3_); ^13^C NMR (101 MHz, CDCl_3_) δ 151.8, 150.7, 150.0, 148.9, 145.1, 134.3, 132.3, 131.0, 129.9, 128.3, 127.3, 126.3, 126.2, 123.2, 121.6, 96.8, 88.9, 54.639.2, 35.8, 31.9, 30.4, 22.7, 14.0; UV–vis (CHCl_3_) λ_max_ [nm] (log ε): 434 (6.06), 564 (4.28), 610 (4.20); IR (neat)/cm^−1^) ν̃: 2921 (m), 2851 (m), 1451 (m), 1305 (m), 1211 (m), 1072 (m), 1008 (s), 939 (m), 787 (s), 750 (s), 708 (s), 639 (s); HRMS–MALDI (*m*/*z*): [M]^+^ calcd for C_100_H_90_N_8_Zn_2_, 1530.5871; found, 1530.5919.

#### Synthesis of [5,15-dihexyl-10-phenyl-20-{(10'-(triisopropylsilyl)ethynyl)-9'-ethynyltriptycenyl}porphyrinato]zinc(II) (**14**)

Synthesized by modified general procedure 1 from porphyrin **13** (20 mg, 29 μmol), triptycene **12** (20 mg, 44 μmol), PdCl_2_(PPh_3_)_2_ (3 mg, 4.35 μmol) and CuI (1 mg, 8.7 μmol) in THF and NEt_3_ (0.75 mL:0.25 mL). The reaction was allowed to stir at 70 °C for four hours. The crude material was purified by filtration through a short silica gel column using *n*-hexane/CH_2_Cl_2_ (4:1, v/v). Recrystallization (CHCl_3_/*n*-hexane) yielded purple crystals (19 mg, 18 μmol, 60%). Mp 296 °C. *R*_f_ = 0.65 (SiO_2_, CH_2_Cl_2_/*n*-hexane, 4:1, v/v); ^1^H NMR (600 MHz, CDCl_3_) δ 10.07 (d, *J* = 4.6 Hz, 2H, *H*_β_), 9.60 (d, *J* = 4.6 Hz, 2H, *H*_β_), 9.45 (d, *J* = 4.6 Hz, 2H, *H*_β_), 8.92 (d, *J* = 4.6 Hz, 2H, *H*_β_), 8.49 (d, *J* = 7.4 Hz, 3H, triptycene-*H*), 8.21–8.19 (m, 2H, phenyl-*H*), 7.98 (d, *J* = 7.4 Hz, 3H, triptycene-*H*), 7.82–7.74 (m, 3H, phenyl-*H*), 7.31 (td, *J* = 7.3, 1.0 Hz, 3H, triptycene-*H*), 7.27 (td, *J* = 7.5, 1.2 Hz, 3H, triptycene-*H*), 4.94–4.87 (m, 4H, hexyl-C*H*_2_), 2.54 (dt, *J* = 15.2, 7.6 Hz, 4H, hexyl-C*H*_2_), 1.85 (dt, *J* = 15.2, 7.6 Hz, 4H, hexyl-C*H*_2_), 1.54 (d, *J* = 7.6 Hz, 4H, hexyl-C*H*_2_), 1.43 (dd, *J* = 7.6, 2.5 Hz, 4H, hexyl-C*H*_2_), 1.40 (dd, 21H, TIPS-C*H*_3_), 0.95 ppm (t, *J* = 7.6 Hz, 6H, hexyl-C*H*_3_); ^13^C NMR (151 MHz, CDCl_3_) δ 151.9, 150.8, 150.1, 149.3, 144.7, 144.1, 142.9, 134.4, 132.8, 132.3, 131.3, 130.2, 128.8, 127.7, 126.7, 126.2, 123.0, 122.8, 122.1, 101.6, 98.1, 95.9, 94.4, 89.9, 54.4, 53.9, 39.1, 35.8, 32.1, 30.5, 29.8, 22.9, 19.0, 14.3, 11.7 ppm; IR (neat)/cm^−1^) ν̃: 2921 (m), 2851 (m), 1451 (m), 1305 (m), 1211 (m), 1072 (m), 1008 (s), 939 (m), 787 (s), 750 (s), 708 (s), 639 (s); UV–vis (CHCl_3_) λ_max_ [nm] (log ε): 430 (5.84), 563 (4.30), 608 (4.35); HRMS–MALDI (*m*/*z*): [M]^+^ calcd for C_71_H_72_N_4_SiZn, 1072.4818; found, 1072.4800.

#### Synthesis of 9-[(10,20-dihexyl-15-phenylporphyrinato-5-yl)ethynyl]zinc(II)-10-[(10,20-dihexyl-15-phenylporphyrinato-5-yl)ethynyl]nickel(II)-triptycene (**16**)

Porphyrin **15** (15.6 mg, 2.2 μmol) and the triptycene–porphyrin **14** (14 mg, 15 μmol) were placed in an oven dried Schlenk flask and heated under vacuum. The flask was purged with argon and anhydrous THF/NEt_3_ (1 mL:0.33 mL) were added by syringe. Argon was bubbled through the solution for five minutes. PdCl_2_(PPh_3_)_2_ (1.5 mg, 2 μmol) and CuI (1 mg, 4 μmol) were added and the reaction mixture was stirred at 70 °C for 18 hours and then diluted with CH_2_Cl_2_ (10 mL) before removal of solvents in vacuo*.* The crude material was purified by filtration with a silica gel column using 2:1: CH_2_Cl_2_/*n*-hexane. Recrystallization (CHCl_3_/*n*-hexane) yielded purple crystals (2 mg, 8%). Mp 335–338 °C. *R*_f_ = 0.7 (SiO_2_, CH_2_Cl_2_/*n*-hexane, 2:1, v/v); ^1^H NMR (600 MHz, CDCl_3_) δ 10.23 (d, *J* = 4.3 Hz, 4H, *H*_β_), 9.70 (d, *J* = 4.3 Hz, 4H, *H*_β_), 9.49 (d, *J* = 4.3 Hz, 4H, *H*_β_), 8.95 (d, *J* = 4.4 Hz, 4H, *H*_β_), 8.70 (dd, *J* = 5.2, 3.3 Hz, 6H, triptycene-*H*), 8.23 (d, *J* = 6.7 Hz, 4H, phenyl-*H*), 7.78 (t, *J* = 6.7 Hz, 6H, phenyl-*H*), 7.48 (dd, *J* = 5.6, 2.4 Hz, 6H, triptycene-*H*), 5.00–4.95 (m, 8H, hexyl-C*H*_2_), 2.63–2.55 (m, 8H, hexyl-C*H*_2_), 1.92–1.84 (m, 8H, hexyl-C*H*_2_), 1.60–1.55 (m, 8H, hexyl-C*H*_2_), 1.48–1.41 (m, 8H, hexyl-C*H*_2_), 0.97 (t, *J* = 7.3 Hz, 12H); ^13^C NMR (101 MHz, CDCl_3_) δ 167.7, 150.6, 150.3, 143.0, 138.5, 134.9, 133.3, 133.1, 128.9, 128.5, 126.8, 120.5, 105.1, 52.5; UV–vis (CHCl_3_) λ_max_ [nm] (log ε): 433 (5.77), 563 (4.32), 609 (4.31); HRMS–MALDI (*m*/*z*): [M]^+^ calcd for C_100_H_90_N_8_NiZn, 1524.5933; found, 1524.5970.

#### Synthesis of 5,15-bis(3'-methoxycarbonylphenyl)-10,20-dibromoporphyrin (**17b**)

Porphyrin **17a** [42] (100 mg, 0.173 mmol) was dissolved in CHCl_3_ (150 mL) and degassed for 30 minutes. At 0 °C, *N*-bromosuccinimide (34 mg, 0.19 mmol) and pyridine (0.1 mL) were added. The reaction was stirred at room temperature for three hours. The progress of the reaction was monitored by TLC and once all starting material had been consumed the reaction mixture was filtered through silica gel using CH_2_Cl_2_ as eluent. The solvents were removed in vacuo and the crude product was purified by recrystallisation from CHCl_3_/CH_3_OH. The product was obtained as purple crystals (19 mg, 15%). Mp 298 °C (dec.). *R*_f_ = 0.75 (SiO_2_, CH_2_Cl_2_/*n*-hexane, 3:1, v/v); ^1^H NMR (400 MHz, CDCl_3_) δ 9.63 (d, *J* = 4.6 Hz, 4H, *H*_β_), 8.85 (s, 2H, phenyl-*H*), 8.77 (d, *J* = 4.6 Hz, 4H, *H*_β_), 8.52 (d, *J* = 7.7 Hz, 2H, phenyl-*H*), 8.34 (d, *J* = 7.7 Hz, 2H, phenyl-*H*), 7.88 (t, *J* = 7.7 Hz, 2H, phenyl-*H*), 4.01 (s, 6H, ester-C*H*_3_), −2.77 ppm (s, 2H, N*H*); ^13^C NMR (101 MHz, CDCl_3_) δ 167.3, 141.8, 138.5, 134.9, 129.5, 129.2, 127.2, 120.2, 104.2, 52.6; IR (neat)/cm^−1^) ν̃: 2923 (m), 2853 (w), 1726 (s), 1580 (w), 1463 (w), 1436 (m), 1286 (m), 1241 (s), 1190 (m), 1105 (m), 961 (m), 791 (s), 746 (s), 728 (s), 629 (m); UV–vis (CHCl_3_): λ_max_ [nm] (log ε) = 424 (5.51), 523 (4.21), 558 (3.00), 603 (3.72), 660 (3.67); HRMS–MALDI (*m*/*z*): [M]^+^ calcd for C_36_H_24_N_4_O_4_Br_2_, 734.0164; found, 734.0186.

#### Synthesis of [5,15-bis(3'-methoxycarbonylphenyl)-10,20-dibromoporphyrinato]zinc(II) (**17c**)

Synthesized from free base porphyrin **17b** (60 mg, 0.081 mmol), Zn(II)(OAc)_2_·2H_2_O (89 mg, 0.41 mmol in CHCl_3_ and MeOH (50 mL:15 mL) by stirring the porphyrin solution at room temperature for four hours. The reaction process was monitored by TLC and once all the starting material was consumed the reaction mixture was washed with NaHCO_3_, dried over MgSO_4_ and filtered through silica gel using CH_2_Cl_2_ as the eluent. Purple crystals were obtained (60 mg, 92%). Mp 307 °C. *R*_f_ = 0.22 (SiO_2_, CH_2_Cl_2_/*n*-hexane, 3:1, v/v); ^1^H NMR (400 MHz, CDCl_3_) δ 9.65 (d, *J* = 4.7 Hz, 4H, *H*_β_), 8.79 (s, 2H, phenyl-*H*), 8.76 (d, *J* = 4.7 Hz, 4H, *H*_β_), 8.45 (d, *J* = 7.7 Hz, 2H, phenyl-*H*), 8.34 (d, *J* = 7.7 Hz, 2H, phenyl-*H*), 7.83 (t, *J* = 7.7 Hz, 2H, phenyl-*H*), 3.98 ppm (d, 6H, ester-C*H*_3_); ^13^C NMR (101 MHz, CDCl_3_) δ 167.7, 150.6, 150.3, 143.0, 138.5, 134.9, 133.3, 133.1, 128.9, 128.5, 126.8, 120.5, 105.1, 52.5; IR (neat)/cm^−1^) ν̃: 1697 (m), 1430 (w), 1286 (m), 1227 (m), 1022 (m), 997 (s), 791 (s), 755 (s), 732 (s), 696 (m); UV–vis (CHCl_3_) λ_max_ [nm] (log ε): 425 (5.68), 556 (4.34), 596 (3.82); HRMS–MALDI (*m*/*z*): [M]^+^ calcd for C_36_H_22_N_4_O_4_Br_2_Zn, 795.9299; found, 795.9333.

#### Synthesis of [5,15-bis{(10'-((triisopropylsilyl)ethynyl)-9'-triptycenyl)ethynyl}-10,20-bis(3'-methoxycarbonylphenyl)porphyrinato]zinc(II) (**18**)

Dibromoporphyrin **17c** (16 mg, 20 μmol) and triptycene **12** (23 mg, 50 μmol) were placed in an oven-dried Schlenk flask and heated under vacuum. The flask was purged with argon and anhydrous THF/NEt_3_ (1 mL :0.33 mL) were added by syringe. Argon was bubbled through the solution for five minutes. PdCl_2_(PPh_3_)_2_ (2 mg, 3 μmol) and CuI (0.7 mg, 6 μmol) were added and the reaction was allowed to stir at 70 °C for 18 hours and then diluted with CH_2_Cl_2_ (10 mL) before removal of solvents in vacuo*.* The crude material was purified by filtration with silica gel column using CH_2_Cl_2_/*n*-hexane (1:1, v/v). Recrystallization (CHCl_3_/*n*-hexane) gave green crystals (6 mg, 19%). Mp 307 °C. *R*_f_ = 0.15 (SiO_2_, CH_2_Cl_2_/*n*-hexane, 2:1, v/v); ^1^H NMR (400 MHz, CDCl_3_) δ 10.12 (d, *J* = 4.4 Hz, 4H, *H*_β_), 9.02 (d, *J* = 4.4 Hz, 4H, *H*_β_), 8.94 (s, 2H, phenyl-*H*), 8.49 (dd, *J* = 18.6, 7.7 Hz, 4H, phenyl-*H*), 8.41 (d, *J* = 6.2 Hz, 6H, triptycene-*H*), 7.96 (d, *J* = 6.2 Hz, 6H, triptycene-*H*), 7.91 (d, *J* = 7.7 Hz, 2H, phenyl-*H*), 4.00 (s, 6H, ester-C*H*_3_), 1.37 ppm (d, 42H, TIPS-C*H*_3_); ^13^C NMR (101 MHz, CDCl_3_) δ 167.3, 152.7, 150.3, 144.3, 143.9, 143.4, 143.1, 142.4, 138.2, 134.7, 133.2, 131.8, 130.9, 129.2, 128.9, 128.8, 127.0, 126.0, 122.7, 101.2, 94.4, 68.5, 54.2, 53.7, 52.4, 37.1, 33.5, 32.2, 31.9, 31.2, 29.7, 29.4, 26.4, 22.6, 19.8, 18.9, 14.4, 14.1, 11.5; IR (neat)/cm^−1^) ν̃: 2926 (w), 1723 (w), 1452 (w), 1260 (m), 1017 (s), 1249 (m), 794 (s), 751 (s), 689 (m), 668 (w), 670 (w); UV–vis (CHCl_3_) λ_max_ [nm] (log ε): 436 (5.92), 573 (4.46), 616 (4.75); HRMS–MALDI (*m*/*z*): [M]^+^ calcd for C_102_H_88_N_4_O_4_Si_2_Zn, 1552.5636; found, 1552.5616.

### Crystal structure determinations

Crystals were grown following the protocol developed by Hope by dissolving the compound in CH_2_Cl_2_ and layering with a MeOH for liquid–liquid diffusion [52]. Single crystal X-ray diffraction data for all compounds were collected on a Bruker APEX 2 DUO CCD diffractometer by using graphite-monochromated Mo K_α_ (λ = 0.71073 Å) radiation. Crystals were mounted on a MiTeGen MicroMount and collected at 100(2) K by using an Oxford Cryosystems Cobra low-temperature device. Data were collected by using omega and phi scans and were corrected for Lorentz and polarization effects by using the APEX software suite [53–55]. Using Olex2, the structures were solved with the XT structure solution program, using the intrinsic phasing solution method and refined against |F2| with XL using least-squares minimization [56–57]. Hydrogen atoms were generally placed in geometrically calculated positions and refined using a riding model. All images were rendered using Olex2.

**Crystal Data for 5:** C_18_H_21_Si (*M* =265.44 g/mol): triclinic, space group P−1 (no. 2), *a* = 10.9626(4) Å, *b* = 11.8640(4) Å, *c* = 12.8821(5) Å, α = 90.244(2)°, β = 93.352(2)°, γ = 98.157(2)°, *V* = 1655.53(10) Å^3^, *Z* = 4, *T* = 100(2) K, μ(Cu K*_α_*) = 1.112 mm^−1^, *D*_calc_ = 1.065 g/cm^3^, 36162 reflections measured (6.874° ≤ 2θ ≤ 133.814°), 5788 unique (*R*_int_ = 0.0696, *R*_sigma_ = 0.0442) which were used in all calculations. The final *R*_1_ was 0.0808 (I > 2σ(I)) and *wR*_2_ was 0.2531 (all data). The three methyl groups on the TMS moiety was modelled over two position in a 55:45% occupancy. Two of the isopropyl group carbon atoms C24 and C26 were two positions in a 50:50% occupancy (see Supporting Information File 2 for full details).

**Crystal data for 16:** C_100_H_90_N_8_NiZn (*M* =1527.87 g/mol): triclinic, space group P−1 (no. 2), *a* = 10.5794(9) Å, *b* = 12.0811(10) Å, *c* = 31.213(3) Å, α = 90.007(2)°, β = 94.017(2)°, γ = 90.367(2)°, *V* = 3979.4(6) Å^3^, *Z* = 2, *T* = 100(2) K, μ(Mo K_α_) = 0.593 mm^−1^, *D*_calc_ = 1.275 g/cm^3^, 85838 reflections measured (1.308° ≤ 2θ ≤ 50.5°), 14446 unique (*R*_int_ = 0.0383, *R*_sigma_ = 0.0279) which were used in all calculations. The final *R*_1_ was 0.0695 (I > 2σ(I)) and *wR*_2_ was 0.1797 (all data). Both phenyl moieties at C15_1 and C15_2 were modelled over two positions in a 56:44% occupancy using the constrain EADP. The porphyrin ring from C17 to C3 in both residues one and two were modelled over two positions in a 50:50% occupancy using the restraint SADI and the constraint EADP. The hexyl groups at the C20_1 and C20_2 positions were modelled over two positions in a 50:50% occupancy using restraints (SADI, ISOR, DFIX) and the constraint EADP. The structure contained a highly disordered chloroform molecule, however as no reasonable solution could be modelled the solvent molecules were omitted using a squeeze protocol (see Supporting Information File 3 for full details).

CCDC 1976646 and1976647 contain the supplementary crystallographic data for this paper. These data can be obtained free of charge from The Cambridge Crystallographic Data Centre via http://www.ccdc.cam.ac.uk/data_request/cif.

## Supporting Information

File 1 Experimental details, copies of NMR and UV–vis spectra, mass spectrometry analyses and X-ray crystallographic details.

File 2 Crystal structure determination of compound **5**.

File 3 Crystal structure determination of compound **16**.
