# Supplementary material for: Towards triptycene functionalization and triptycene-linked porphyrin arrays
Source: Beilstein J Org Chem. 2020 Apr 17;16:763–77. doi: 10.3762/bjoc.16.70 (PMC7176921; doi:10.3762/bjoc.16.70)
Supplement: File 1 — Experimental details, copies of NMR and UV–vis spectra, mass spectrometry analyses and X-ray crystallographic details. [file Beilstein_J_Org_Chem-16-763-s001.pdf]

BEILSTEIN JOURNAL OF ORGANIC CHEMISTRY

Supporting Information

for

Towards triptycene functionalization and triptycene-linked porphyrin arrays

Gemma M. Locke, Keith J. Flanagan and Mathias O. Senge

Beilstein J. Org. Chem. **2020,** *16,* 763–777. doi:10.3762/bjoc.16.70

Experimental details, copies of NMR and UV–vis spectra, mass spectrometry analyses and X-ray crystallographic details

License and Terms: This is a supporting information file under the terms of the Creative Commons Attribution License (http://creativecommons.org/licenses/by/4.0). Please note that the reuse, redistribution and reproduction in particular requires that the authors and source are credited.
The license is subject to the *Beilstein Journal of Organic Chemistry* terms and conditions: (https://www.beilstein-journals.org/bjoc)

Table of contents

	Page
1. General	S1
2. Synthetic attempts with 9,10-dibromotriptycene	S2
3. Spectroscopic and mass spectrometry characterizations	S6
4. X-ray crystal structure determinations	S23
5. Additional illustrations	S27
6. References	S28

1. General

All commercial chemicals used were supplied by Sigma Aldrich, Acros Organics, Fluka Frontier Scientific, Inc. and Fischer and used without further purification unless otherwise stated. Anhydrous tetrahydrofuran (THF), diethyl ether, and CH_2Cl_2 were withdrawn from an automated solvent purification system. To protect air- and moisture sensitive compounds, the corresponding reactions were carried out under "Schlenk conditions" using argon as inert gas. Air and residual moisture were removed from the instruments by a hot-air gun under high vacuum and the flasks were purged with argon subsequently.

Reactions were monitored by thin layer chromatography (TLC) carried out on silica gel plates using UV light as a visualizing agent. Analytical thin layer chromatography was performed using silica gel 60 (fluorescence indicator F_{254}, precoated sheets, 0.2 mm thick, 20 cm × 20 cm; Merck) or aluminium oxide 60 (neutral, F_{254}; Merck) plates and visualized by UV irradiation (λ = 254 nm). Column chromatography was carried out using silica Gel (230–400 mesh; Merck) or aluminium oxide (neutral, activated with 6% H_2O, Brockman Grade III). Mobile phases are given as (v/v). Yields refer to chromatographically and spectroscopically (^{1}H NMR) homogeneous material, unless otherwise noted. Room temperature refers to 20–25 °C.

Melting points are uncorrected and were measured with a Stuart SMP-50 melting point apparatus. NMR spectra were recorded using Bruker DPX400 (400 MHz for ^{1}H NMR, 101 MHz for ^{13}C NMR), Bruker AV 600 (600 MHz for ^{1}H NMR, 151 MHz for ^{13}C NMR) and Bruker AV 400 (400 MHz for ^{1}H NMR, 101 MHz for ^{13}C NMR) instruments. All NMR experiments were performed at room temperature. Chemical shifts are given in ppm and referenced to the residual peak of the deuterated NMR solvent. Signal multiplicities are abbreviated as follows: singlet = s, multiplet = m, doublet = d, doublet of doublets = dd. The assignment of the signals was confirmed by 2D spectra (COSY, HMBC, HSQC) except for those compounds with low solubility. ESI mass spectra were acquired in positive or negative modes as required, using a Micromass time-of-flight mass spectrometer (TOF), or a Bruker mircoOTOF-Q II spectrometer interfaced to a Dionex UltiMate 3000 LC. APCI experiments were carried out on a Bruker microOTOF-Q III spectrometer interfaced to a Dionex Ultimate 3000 C or direct insertion probe in positive or negative modes. IR spectra were recorded on a PerkinElmer Spectrum 100 FT-IR spectrometer. UV–vis spectra were recorded in

solutions using a Specord 250 spectrophotometer from Analytik Jena (1 cm path length quartz cell). Emission, excitation spectra and lifetimes were measured using a Cary Eclipse G9800A fluorescence spectrophotometer and Horiba Jobin Yvon Fluorolog 4. *In vacuo*, either refers to use of a Büchi Rotavapor R-200; a Büchi Rotavapor R-210; a Büchi Rotavapor R-100 with a Büchi V-491 heating bath and Büchi V-850 vacuum controller.

2. Synthetic attempts with 9,10-dibromotriptycene

To synthesize linearly linked triptycene porphyrin dimers, the attachment of various linker groups at the triptycene bridgehead carbon atoms was investigated. Initially 9,10-dibromotriptycene **S2** was selected for functionalization through various Pd-catalyzed cross-coupling reactions. Thereby, 9,10-dibromotriptycene **S2** itself was synthesized from 9,10-dibromoanthracene (**S1**) via a Diels–Alder reaction with anthranilic acid and isopentyl nitrite in dimethoxyethane (DME).[1] The crude material was purified by flash chromatography using silica gel and *n*-hexane. The desired triptycene product was isolated as a yellow powder in 25% yield. The reason for the low yield obtained is due to the instability of the benzyne reagent that is formed in situ. The initial goal of this project was to expand the library of 9,10-difunctionalized triptycenes **S3** as a platform for further functionalization (Scheme S1). With 9,10-dibromotriptycene now in hand, various small molecules, such as boronic acids, boronic esters and ethynyltrimethylsilane (TMSA) were reacted with **S3** under Sonogashira and Suzuki cross-coupling conditions [2,3].

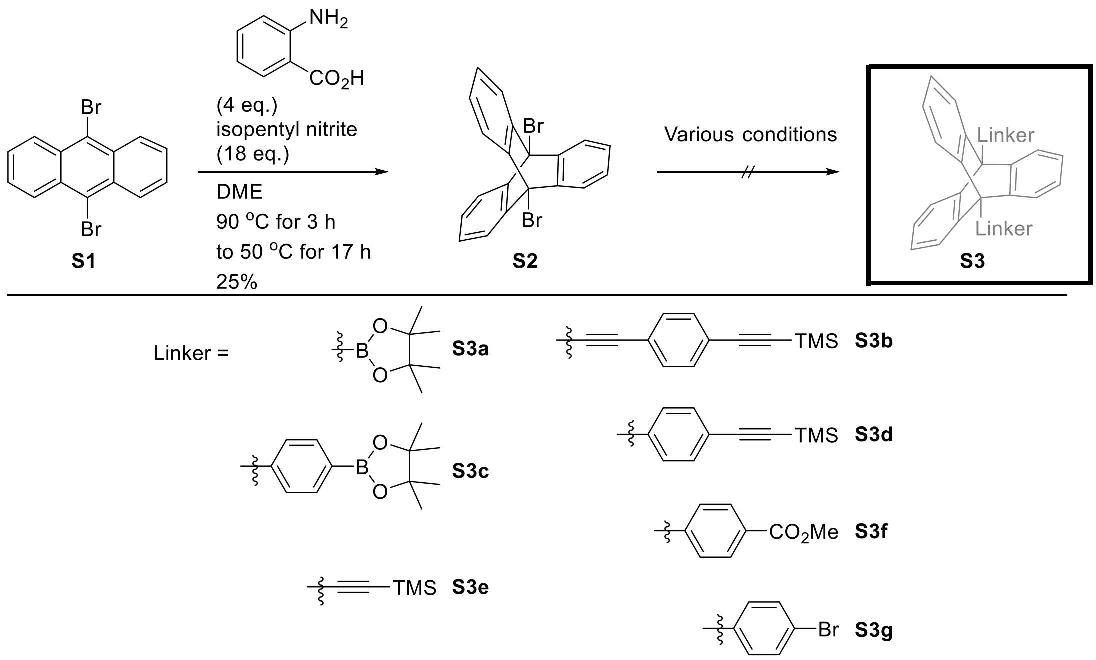

Scheme S1: Synthetic targets to expand the library of linkers available for 9,10-difunctionalized triptycenes.

In an attempt to access 9,10-bis(4-((trimethylsilyl)ethynyl)phenyl)triptycene **S3d**, dibromotriptycene **S2** and the boronic ester **S4** were reacted under Suzuki cross-coupling conditions (Scheme S2) [2]. The two reagents were dried in a Schlenk tube, along with the base K_3PO_4. THF was added and the solution was saturated with argon for 5−10 minutes. The catalyst $Pd(PPh_3)_4$ was then added and the solution was stirred at 70 °C for 18 hours. The crude product was purified *via* silica gel column chromatography (*n*-hexane:CH_2Cl_2, 1:1, v/v). The various isolated fractions required further purification. Therefore, the solvent was removed in vacuo and the crude material was purified using a preparative TLC plate (Et_2O/*n*-hexane, 2:1, v/v). The main product gave a ^{1}H NMR spectrum with only the characteristic triptycene peaks observed at 7.83 and 7.14 ppm while no signals were observed for the phenyl ring or TMS unit of the boronic ester **S4** indicating that the desired product had not been formed.

Following the failed Suzuki cross-coupling reaction, another experiment was performed to synthesize a different 9,10-difunctionalized triptycene, this time using Sonogashira cross-coupling conditions.[3] 9,10-Dibromotriptycene **S2** was reacted with TMSA in order to obtain 9,10-bis((trimethylsilyl)ethynyl)triptycene **S3e** (Scheme S2). In a Schlenk tube, the anthracene **S1** was added and dried for two hours. The

reagent was then dissolved in THF and NEt_3 and the solution was bubbled with argon for 5−10 minutes. The catalyst $Pd(PPh_3)_2Cl_2$ was added alongside the reagent TMSA. The reaction was stirred at 70 °C under argon for 18 hours. TLC analysis showed the presence of a new product with the same colour and R_f as the desired product **S3e**. But again, ^{1}H NMR and mass spectrometry did not indicate product formation but rather the retention and decomposition of the starting material **S2**.

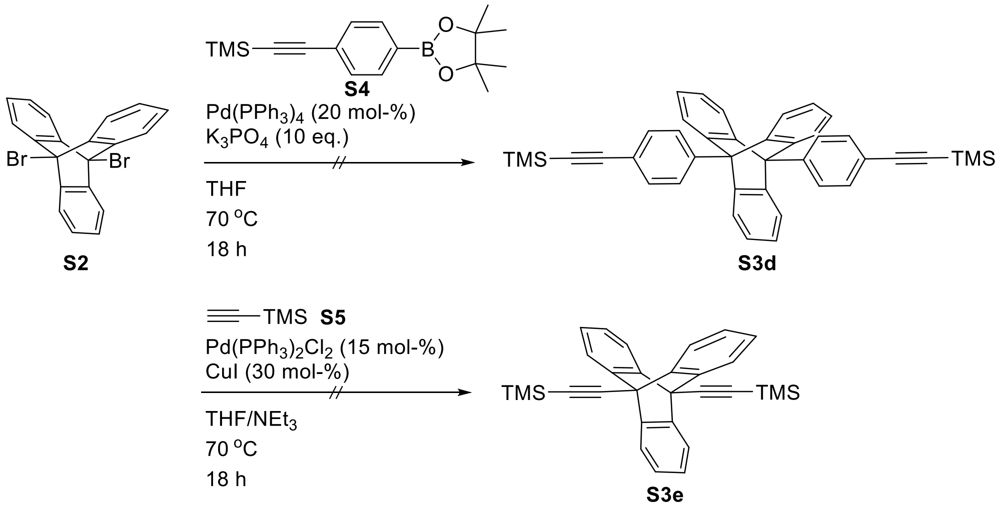

Scheme S2: Suzuki and Sonogashira cross-coupling reactions with 9,10-dibromotriptycene.

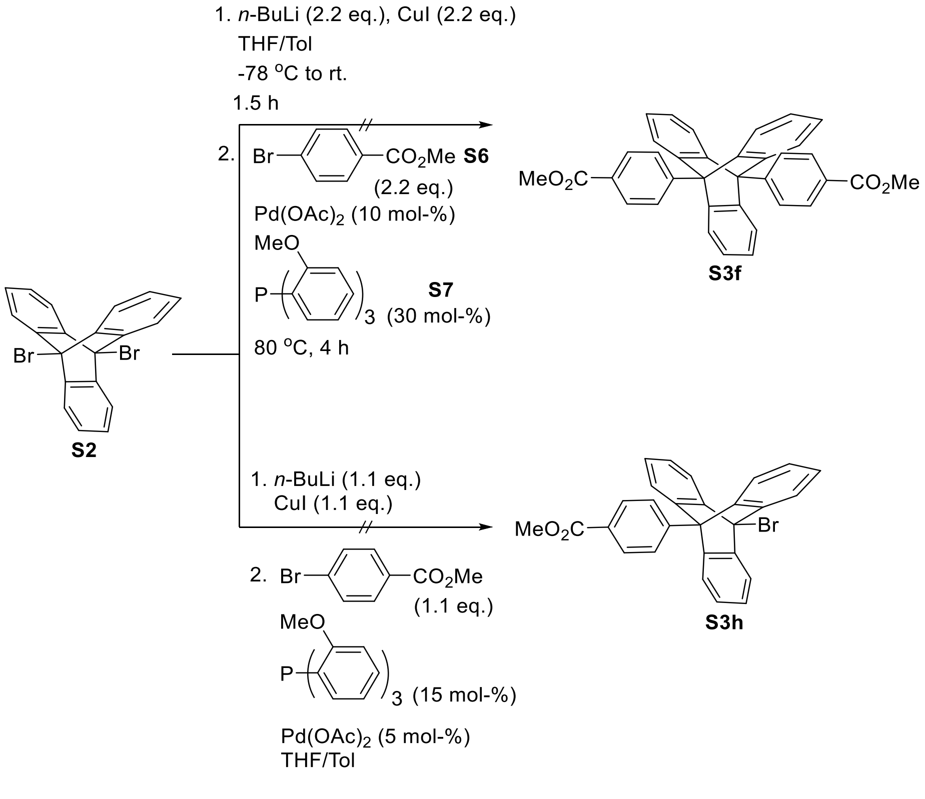

Scheme S3: Organocopper cross-coupling reactions with 9,10-dibromotriptycene **S2**.

Owing to the lack of results produced by the Pd-catalyzed cross-coupling route, a different method was sought in order to functionalize 9,10-dibromotriptycene. A recent paper by Uchiyama et al. showed the use of organocopper cross-coupling reactions to functionalize the bridgehead carbon atoms of triptycene by using 9-bromotriptycene [4]. While 9-bromotriptycene was utilised in the paper the aim of this work is to obtain disubstituted triptycene products, consequently the methods outlined in the publication were applied to 9,10-dibromotriptycene **S2** (Scheme S3). The reaction was performed with twice the equivalents of the reagents to functionalize both bridgehead positions of the triptycene. In order to form the organocopper reagent, triptycene **S2** was reacted with *n*-BuLi followed by the addition of CuI. The catalyst $Pd(OAc)_2$, along with the ligand tris(2-methoxyphenyl)phosphine and reagent methyl 4-bromobenzoate were then added to the reaction mixture and after 18 hours a brown solution was observed. Purification of the reaction mixture was attempted but numerous products were observed through TLC analysis. Due to the impurity of the sample, inconclusive ^{1}H NMR and mass spectrometer analysis of the product **S3f** no further purification was carried out.

Following this, as the literature procedure [4] uses a monobrominated triptycene, the reaction was performed again with triptycene **S2** but with only one equivalent of *n*-BuLi, CuI and the phenyl ester (Scheme S3). Initial purification and TLC analysis showed the presence of several different products, but again mass spectrometry analysis did not show the formation of the desired product **S3h**.

3. Spectroscopic and mass spectrometry characterizations

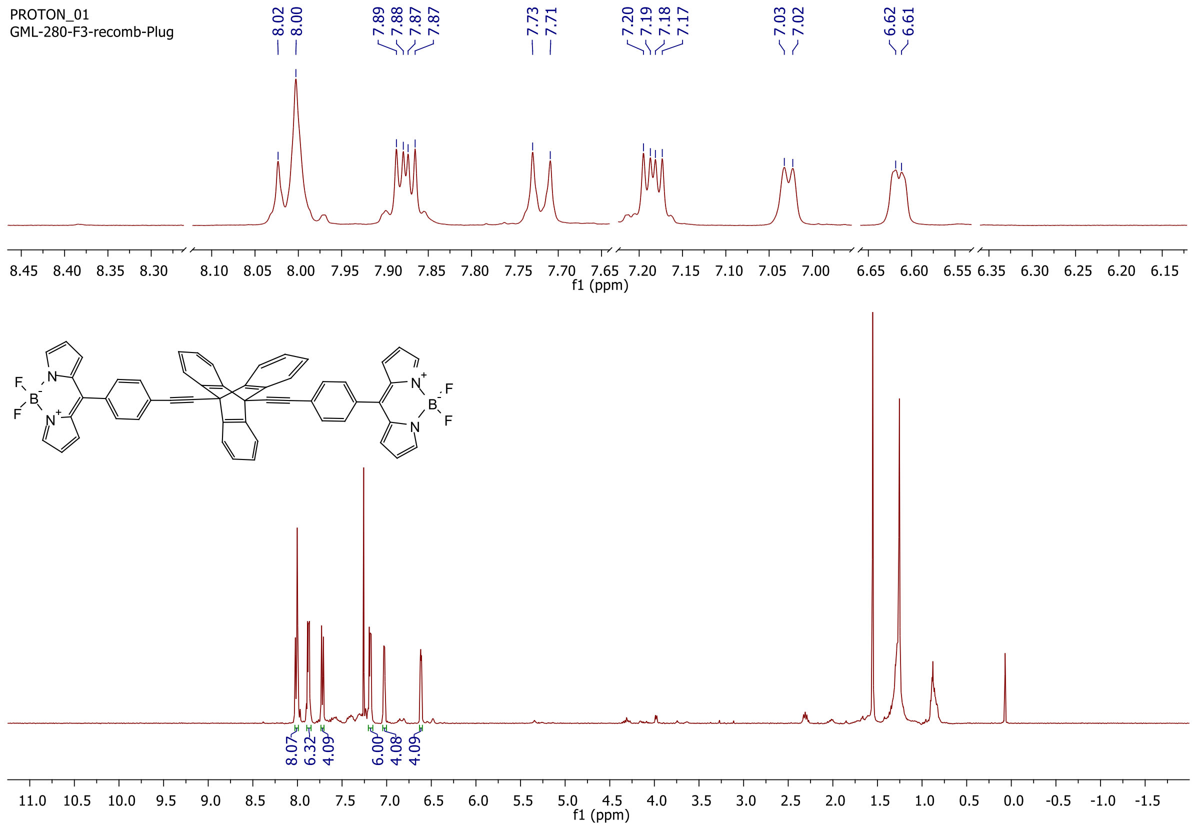

Figure S1: ^{1}H NMR of compound **7** in $CDCl_3$.

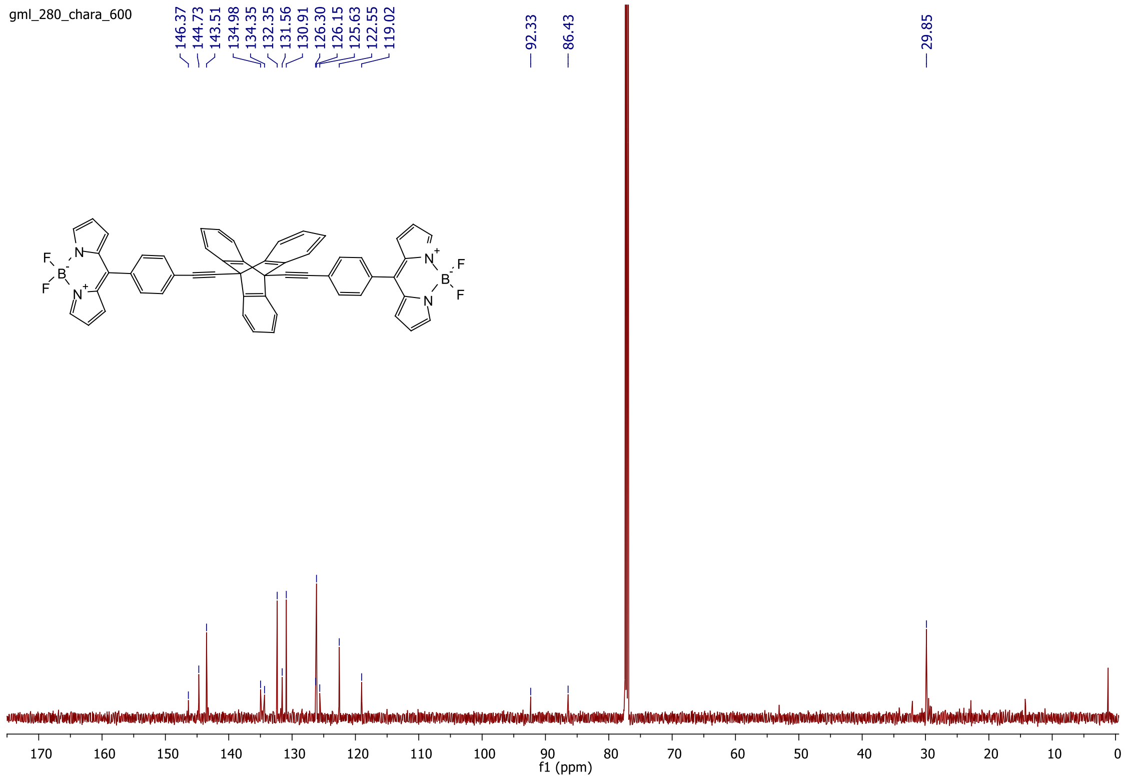

Figure S2: ^{13}C NMR of compound **7** in $CDCl_3$.

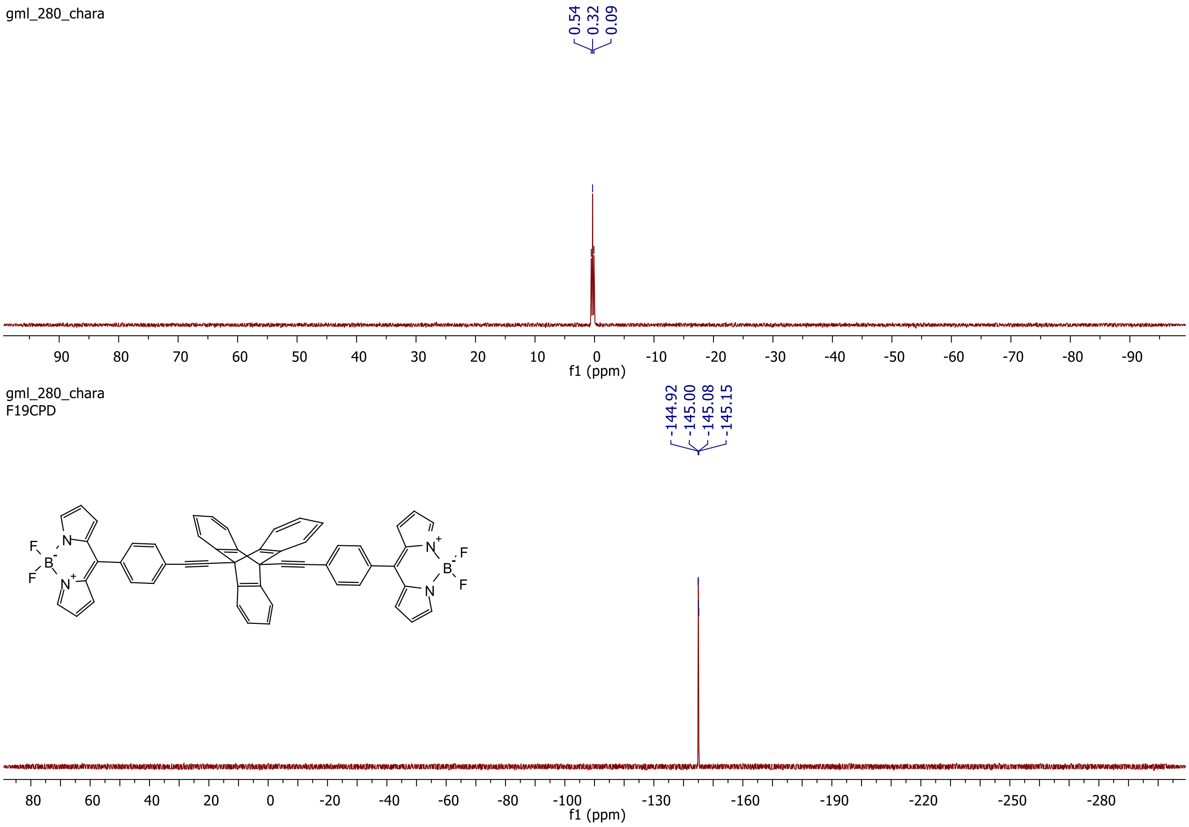

Figure S3: ^{11}B and ^{19}F NMR of compound **7** in $CDCl_3$.

Elemental Composition Report **Page 1**

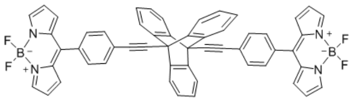

Single Mass Analysis
Tolerance = 50.0 PPM / DBE: min = -1.5, max = 400.0
Element prediction: Off
Number of isotope peaks used for i-FIT = 5

Monoisotopic Mass, Odd and Even Electron Ions
28 formula(e) evaluated with 1 results within limits (up to 10 best isotopic matches for each mass)
Elements Used:
C: 0-54 H: 0-32 N: 0-4 F: 0-4 B: 0-2

Gemma Locke (MSe), GML-280-F3
Q-TOF20180724MF012 15 (0.278) AM (Cen,6, 80.00, Ht,10000.0,0.00,0.70); Sm (SG, 50x3.00); Sb (15,10.00); Cm (10:43) TOF MS LD+ 3.95e+002

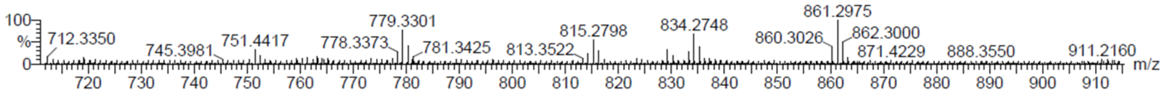

	Mass	Calc. Mass	mDa	PPM	DBE	i-FIT	i-FIT (Norm)	Formula
Minimum:					-1.5			
Maximum:			5.0	50.0	400.0			
	834.2748	834.2749	-0.1	-0.1	40.0	78.5	0.0	C54 H32 N4 F4 B2

Figure S4: Mass spectrum of compound **7** in $CHCl_3$.

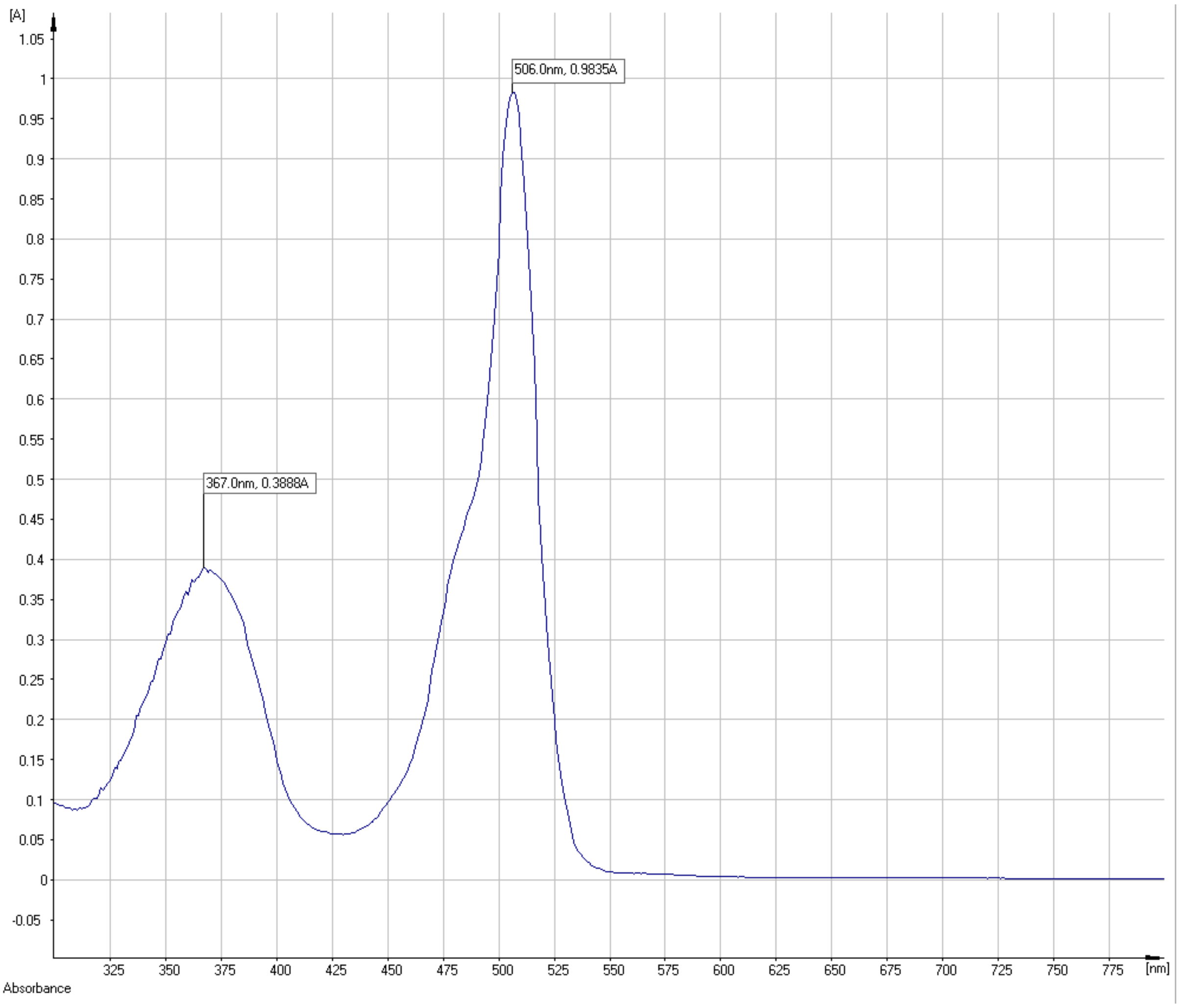

Figure S5: UV–vis spectrum of compound **7** in $CHCl_3$.

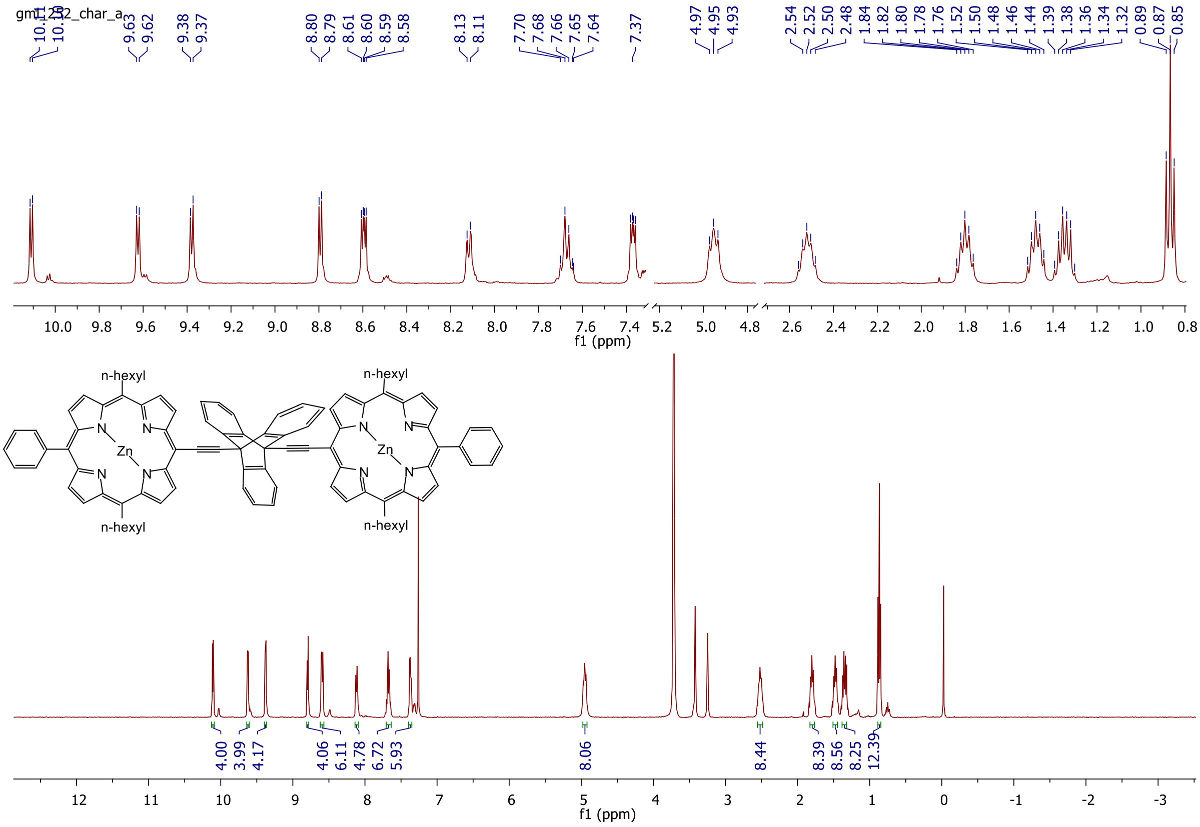

Figure S6: ^{1}H NMR of compound **9** in $CDCl_3$.

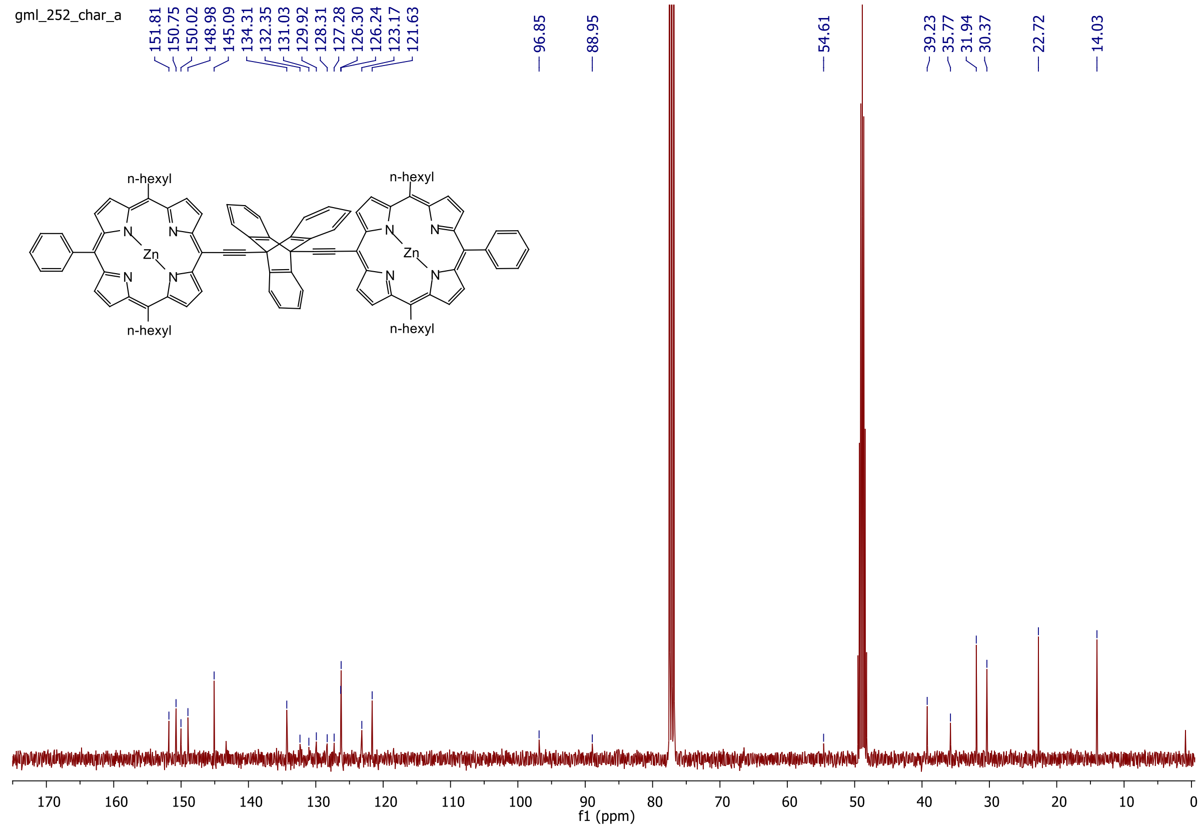

Figure S7: ^{13}C NMR of compound **9** in $CDCl_3$.

Elemental Composition Report

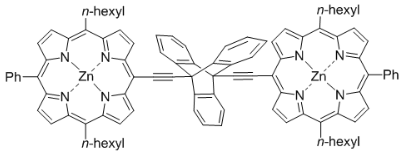

Page 1

Single Mass Analysis
Tolerance = 10.0 PPM / DBE: min = -1.5, max = 100.0
Element prediction: Off
Number of isotope peaks used for i-FIT = 5

Monoisotopic Mass, Odd and Even Electron Ions
13 formula(e) evaluated with 1 results within limits (up to 10 best isotopic matches for each mass)
Elements Used:
C: 0-100 H: 0-90 N: 0-8 Zn: 0-2
Gemma Locke (MSe), GML-232
Q-TOF20171129MF001 24 (0.442) AM (Cen,4, 80.00, Ht,10000.0,1570.68,0.70); Sm (SG, 2x3.00); Sb (15,10.00); Cm (3:109-51:68)

TOF MS LD+
1.35e+003

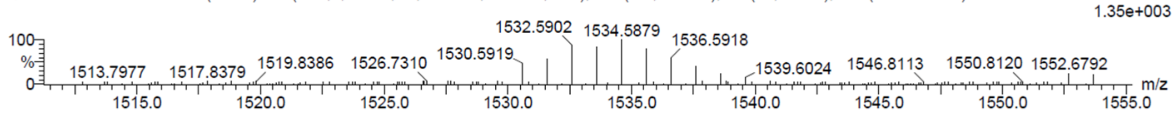

Minimum:				-1.5			
Maximum:		5.0	10.0	100.0			
Mass	Calc. Mass	mDa	PPM	DBE	i-FIT	i-FIT (Norm)	Formula
1530.5919	1530.5871	4.8	3.1	60.0	35.3	0.0	C100 H90 N8 Zn2

Figure S8: Mass spectrum of compound **9** in $CHCl_3$.

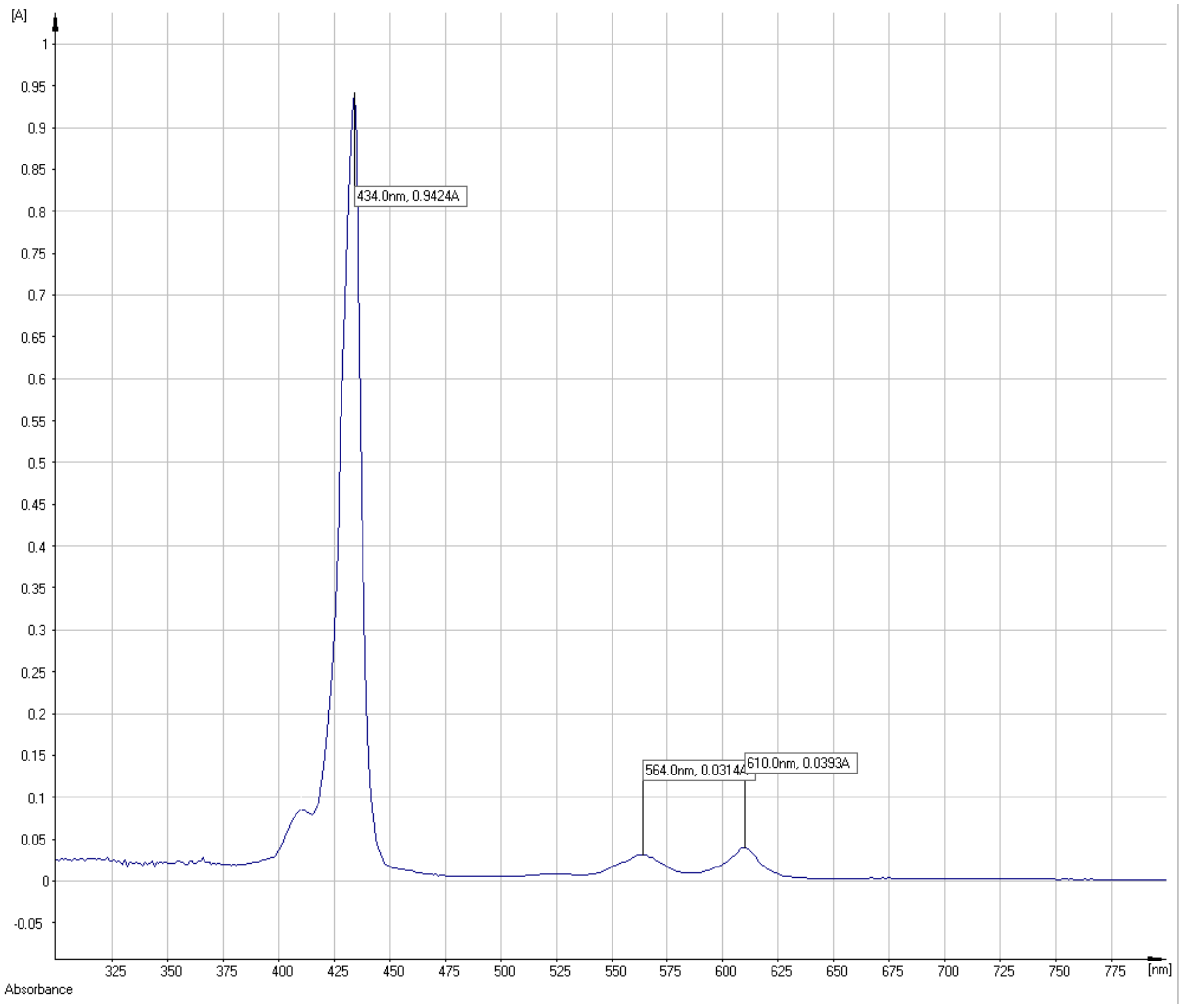

Figure S9: UV–vis spectrum of compound **9** in $CHCl_3$.

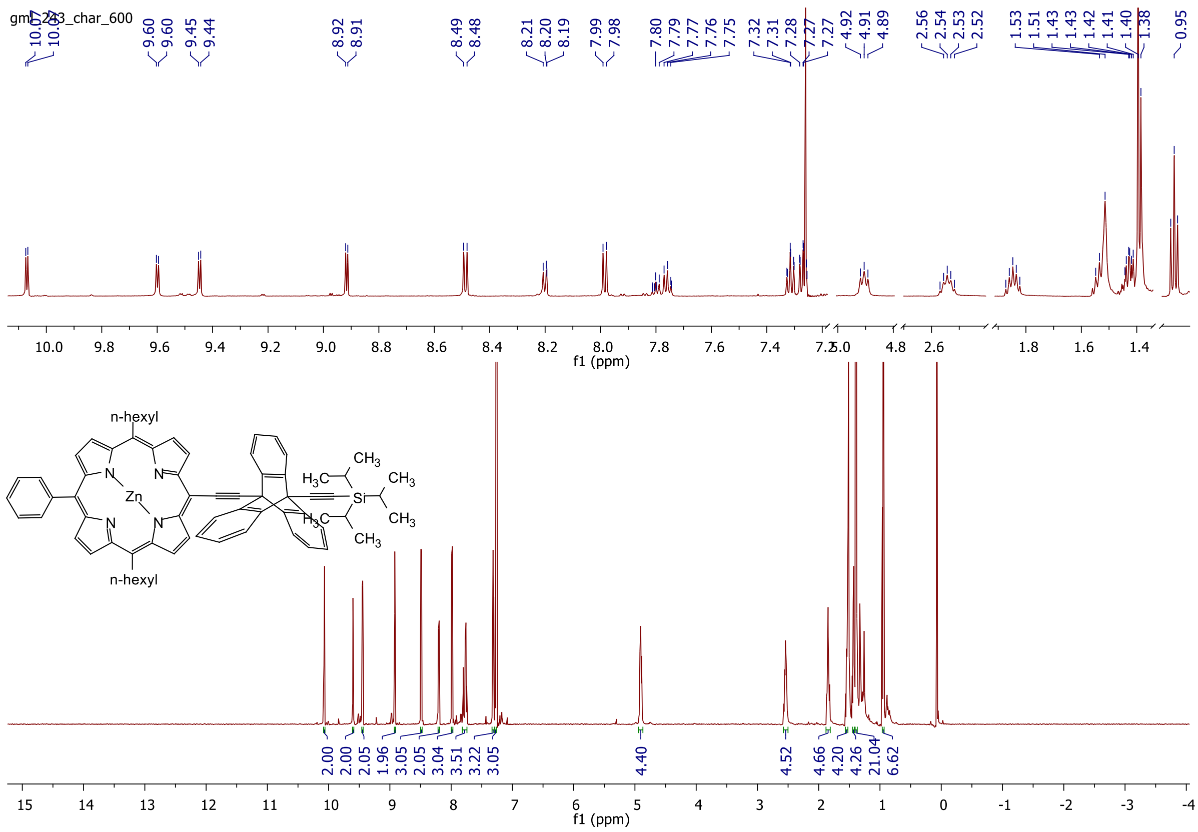

Figure S10: ^{1}H NMR of compound **14** in $CDCl_3$.

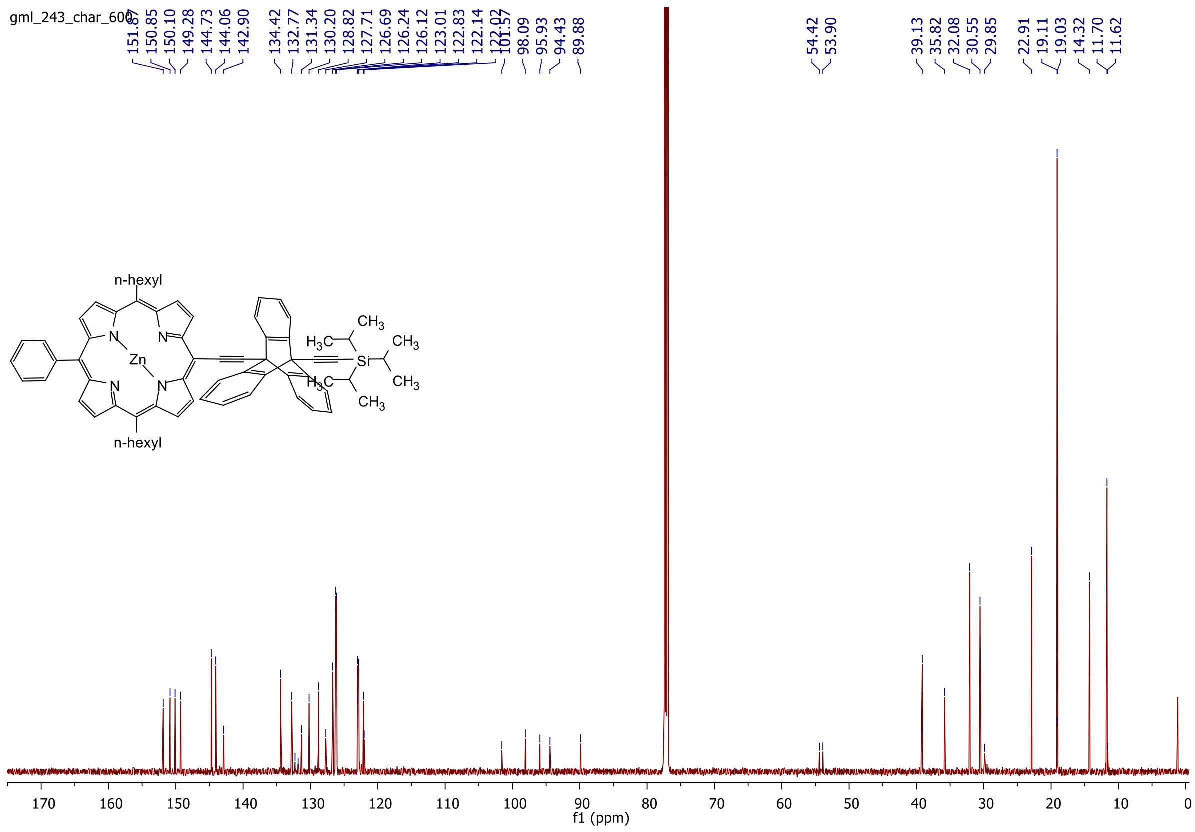

Figure S11: ^{13}C NMR of compound **14** in $CDCl_3$.

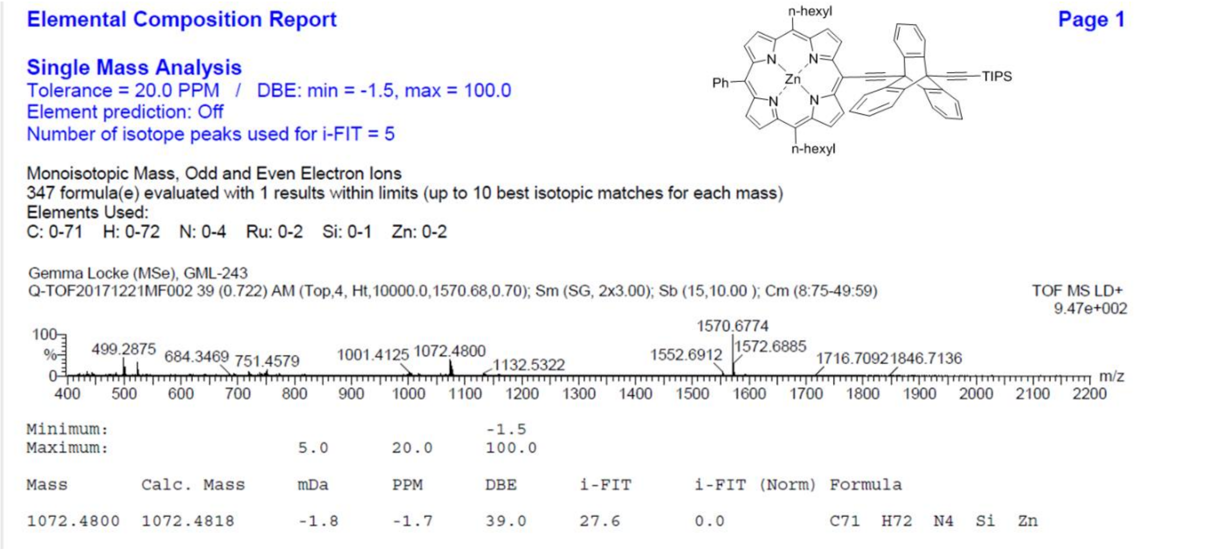

Elemental Composition Report — Page 1

Single Mass Analysis
Tolerance = 20.0 PPM / DBE: min = -1.5, max = 100.0
Element prediction: Off
Number of isotope peaks used for i-FIT = 5

Monoisotopic Mass, Odd and Even Electron Ions
347 formula(e) evaluated with 1 results within limits (up to 10 best isotopic matches for each mass)
Elements Used:
C: 0-71 H: 0-72 N: 0-4 Ru: 0-2 Si: 0-1 Zn: 0-2

Gemma Locke (MSe), GML-243
Q-TOF20171221MF002 39 (0.722) AM (Top,4, Ht,10000.0,1570.68,0.70); Sm (SG, 2x3.00); Sb (15,10.00); Cm (8:75-49:59)
TOF MS LD+
9.47e+002

Minimum:				-1.5			
Maximum:		5.0	20.0	100.0			
Mass	Calc. Mass	mDa	PPM	DBE	i-FIT	i-FIT (Norm)	Formula
1072.4800	1072.4818	-1.8	-1.7	39.0	27.6	0.0	C71 H72 N4 Si Zn

Figure S12: Mass spectrum of compound **14** in $CHCl_3$.

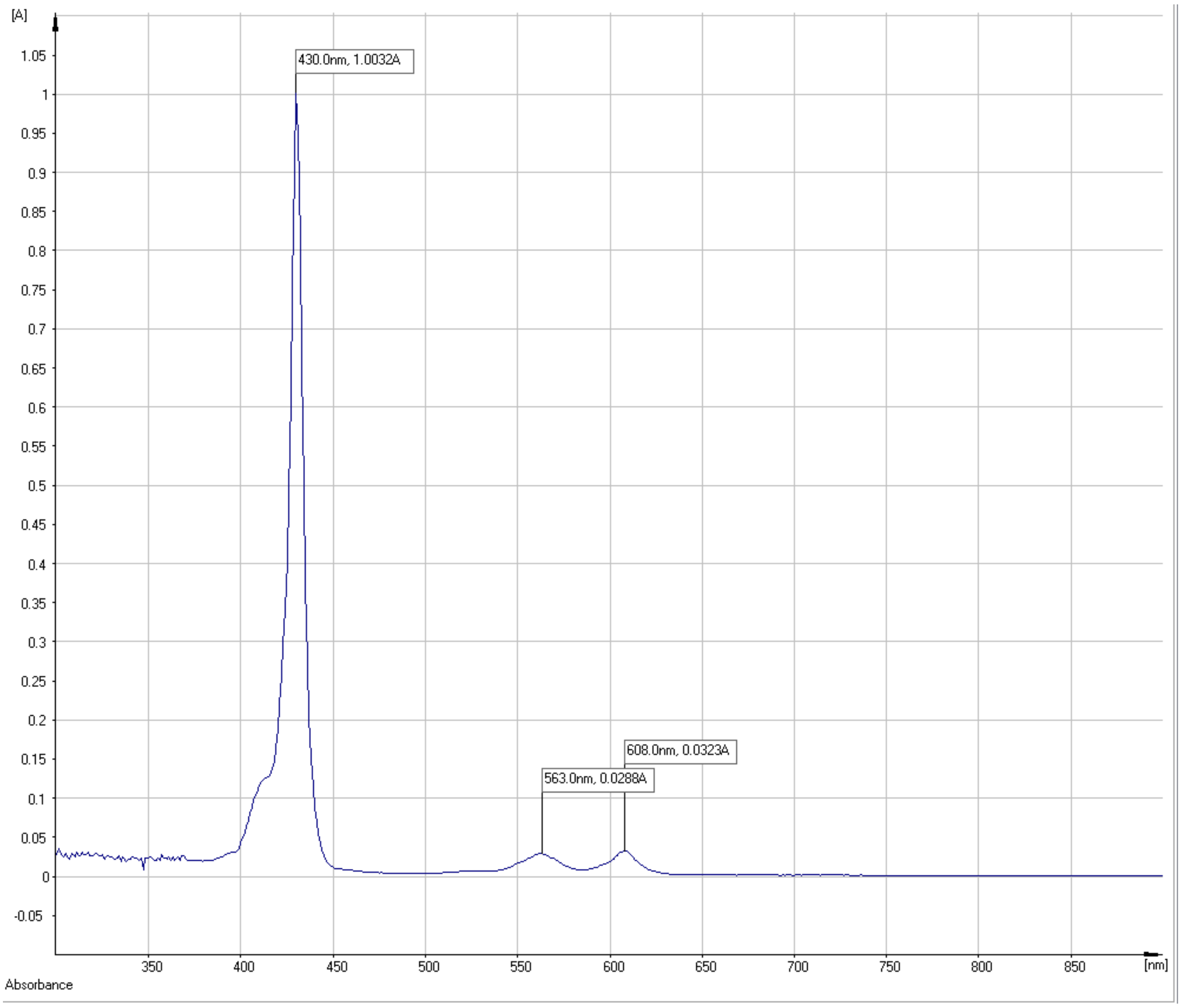

Figure S13: UV–vis spectrum of compound **14** in $CHCl_3$.

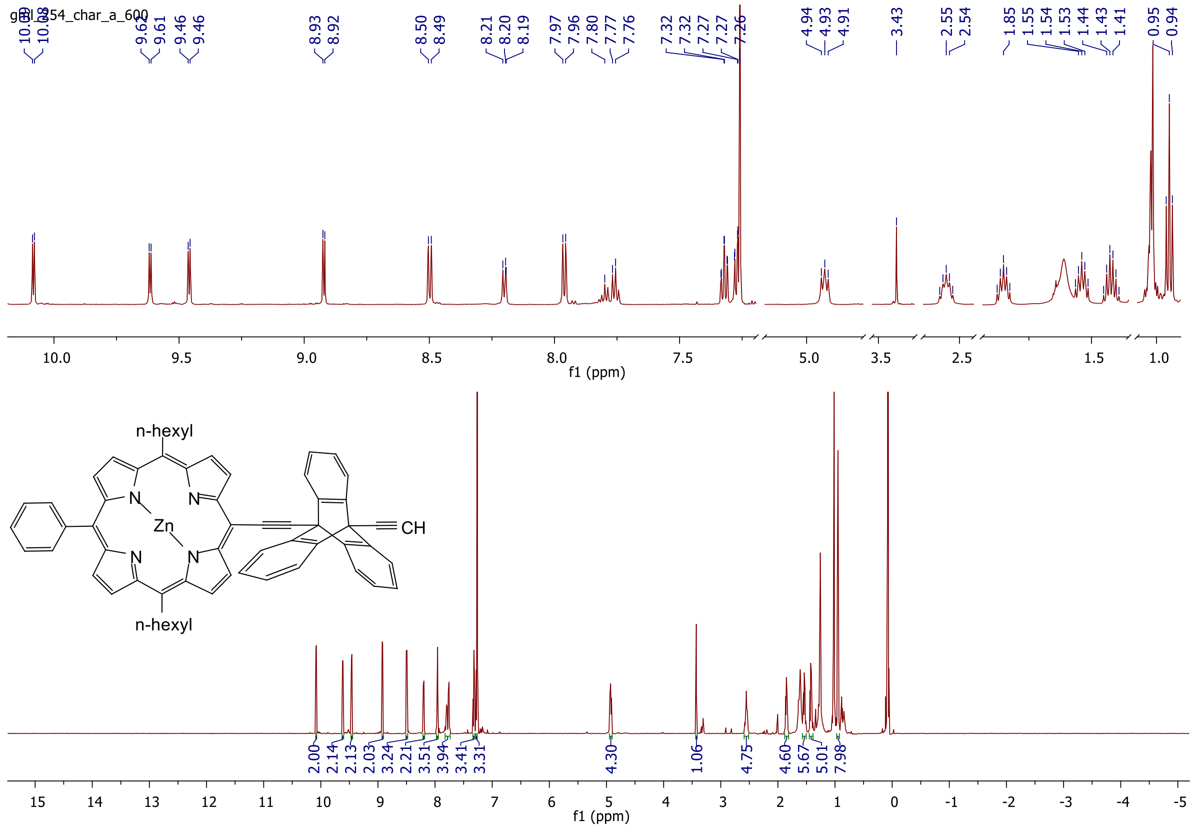

Figure S14: ^{1}H NMR of compound **14a** in $CDCl_3$.

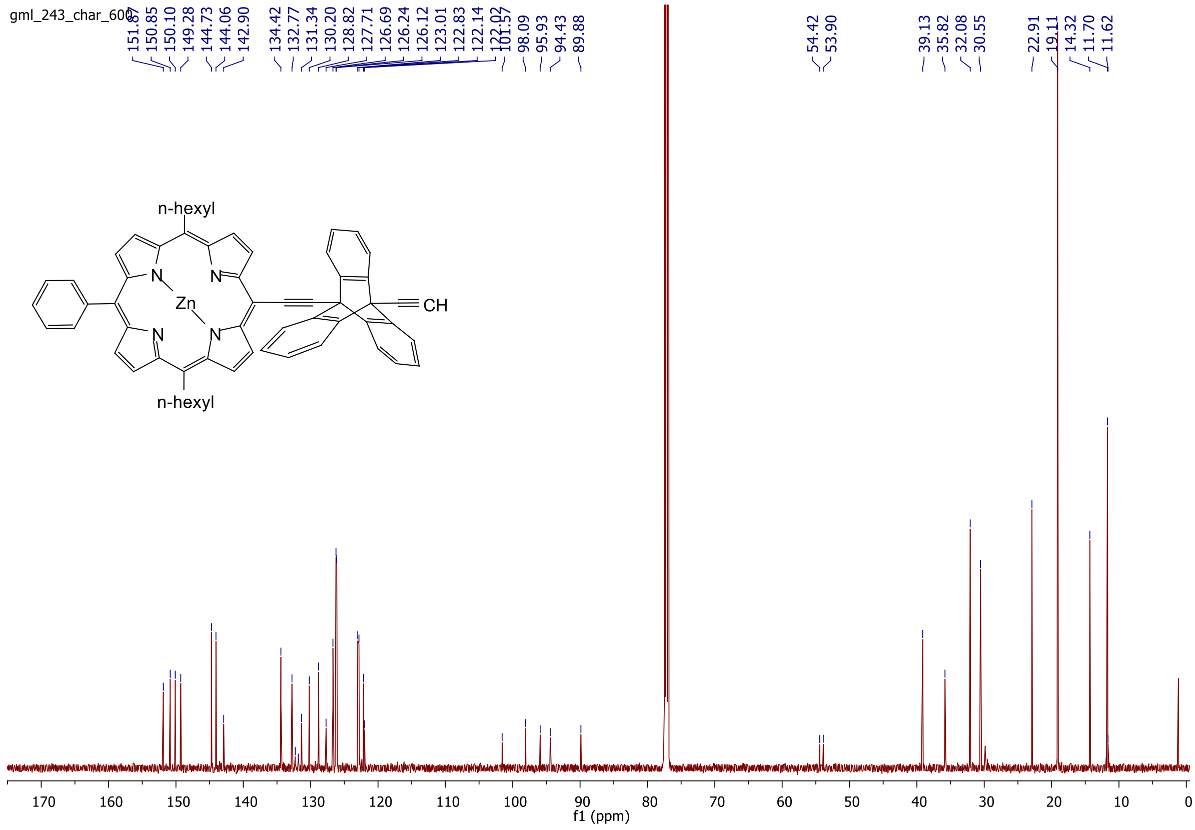

Figure S15: ^{13}C NMR of compound **14a** in $CDCl_3$.

Elemental Composition Report

Page 1

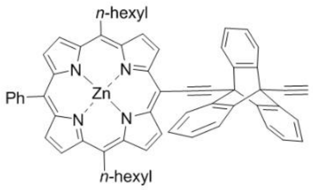

Single Mass Analysis
Tolerance = 50.0 PPM / DBE: min = -1.5, max = 400.0
Element prediction: Off
Number of isotope peaks used for i-FIT = 5

Monoisotopic Mass, Odd and Even Electron Ions
4 formula(e) evaluated with 1 results within limits (up to 10 best isotopic matches for each mass)
Elements Used:
C: 0-62 H: 0-52 N: 0-4 Zn: 0-1

Gemma Locke (MSe), GML-254
Q-TOF20180913MF001 30 (0.556) AM (Cen,6, 80.00, Ht,10000.0,1570.68,0.70); Sm (SG, 2x3.00); Sb (15,10.00); Cm (5:81)

TOF MS LD+
6.90e+002

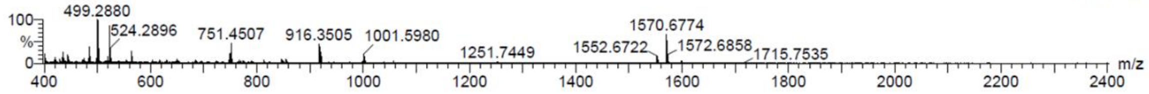

		mDa	PPM	DBE	i-FIT	i-FIT (Norm)	Formula
Minimum:				-1.5			
Maximum:		5.0	50.0	400.0			
Mass	Calc. Mass	mDa	PPM	DBE	i-FIT	i-FIT (Norm)	Formula
916.3505	916.3483	2.2	2.4	39.0	76.5	0.0	C62 H52 N4 Zn

Figure S16: Mass spectrum of compound **14a** in $CHCl_3$.

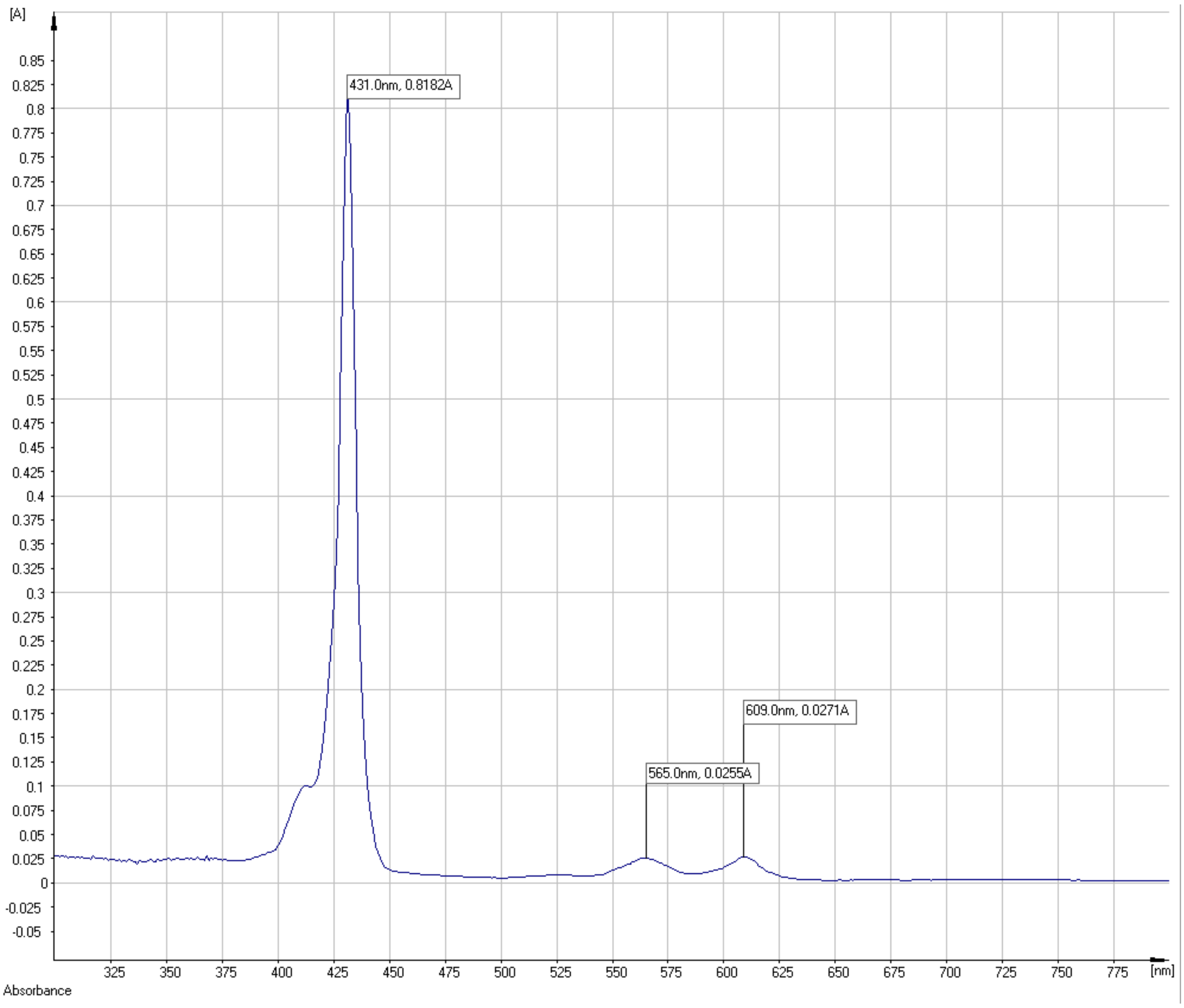

Figure S17: UV–vis spectrum of compound **14a** in $CHCl_3$.

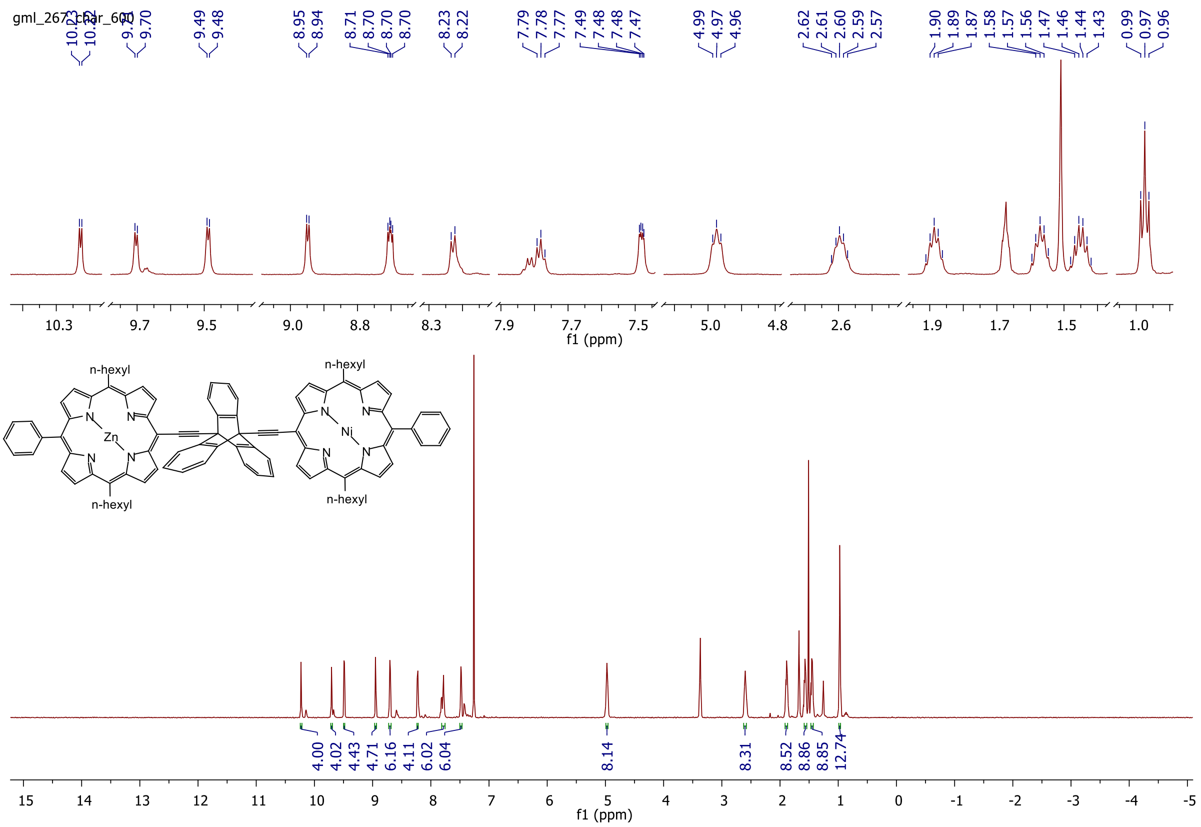

Figure S18: ^{1}H NMR of compound **16** in $CDCl_3$.

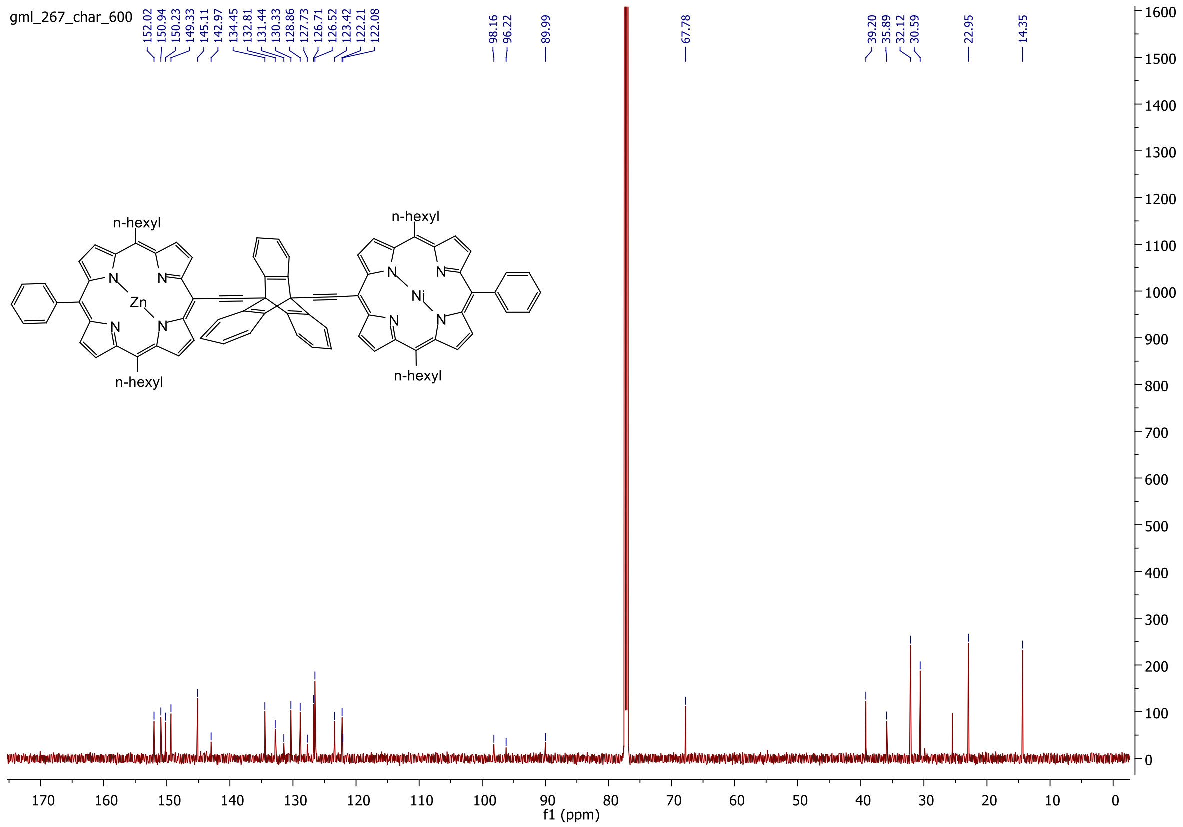

Figure S19: ^{13}C NMR of compound **16** in $CDCl_3$.

Elemental Composition Report

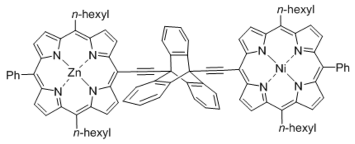

Page 1

Single Mass Analysis
Tolerance = 10.0 PPM / DBE: min = -1.5, max = 200.0
Element prediction: Off
Number of isotope peaks used for i-FIT = 5

Monoisotopic Mass, Odd and Even Electron Ions
5 formula(e) evaluated with 1 results within limits (up to 10 best isotopic matches for each mass)
Elements Used:
C: 0-100 H: 0-90 N: 0-8 Ni: 0-1 Zn: 1-1

Gemma Locke (MSe), GML-267
Q-TOF20180607MF004 35 (0.649) AM (Cen,6, 80.00, Ht,10000.0,1570.68,0.70); Sm (SG, 2x3.00); Sb (15,10.00); Cm (4:94-(53:57+65:68)) TOF MS LD+
7.19e+002

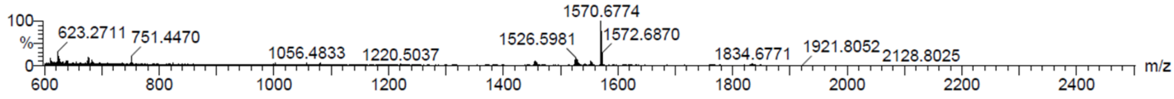

Minimum:				-1.5			
Maximum:		5.0	10.0	200.0			
Mass	Calc. Mass	mDa	PPM	DBE	i-FIT	i-FIT (Norm)	Formula
1524.5970	1524.5933	3.7	2.4	60.5	52.2	0.0	C100 H90 N8 Ni Zn

Figure S20: Mass spectrum of compound **16** in $CHCl_3$.

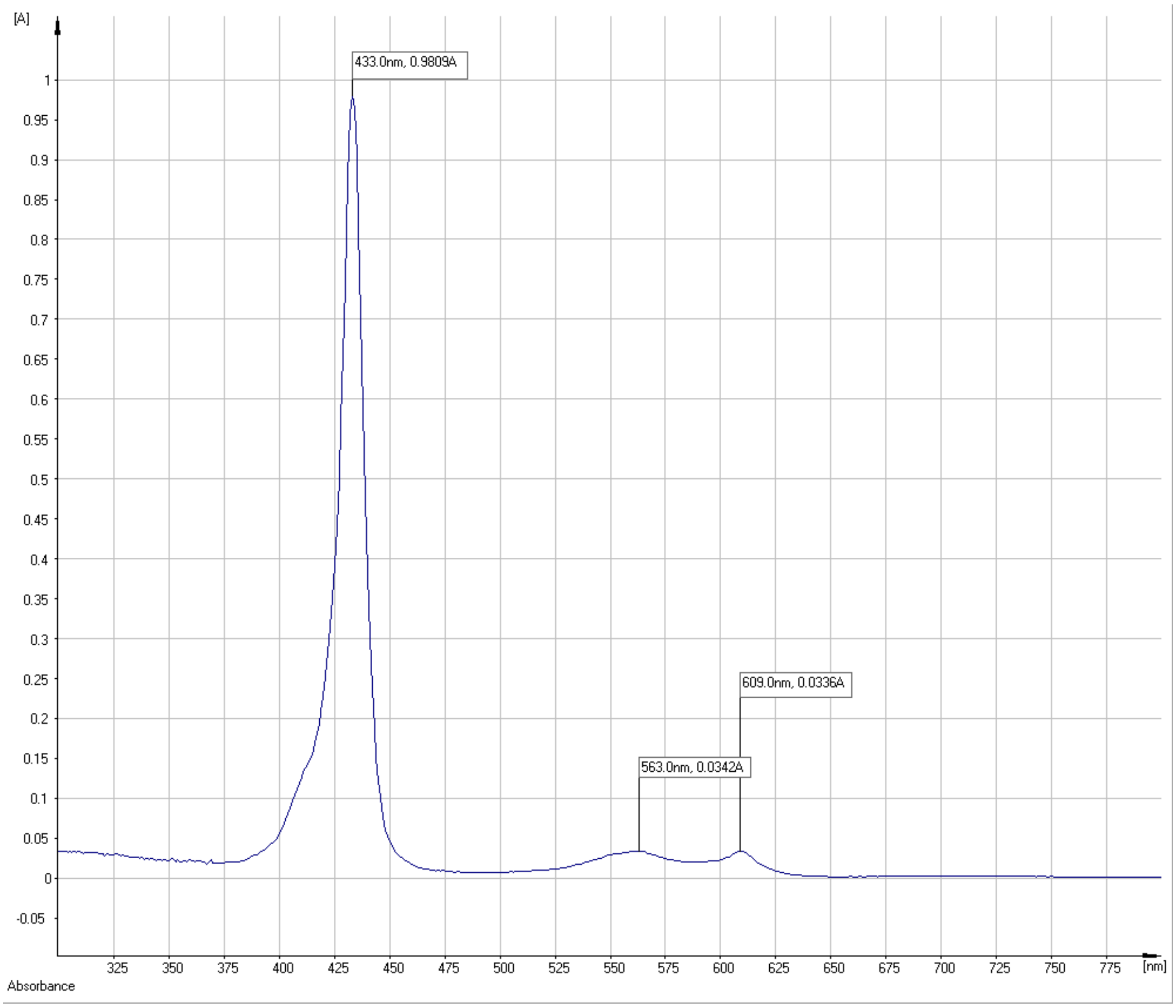

Figure S21: UV–vis spectrum of compound **16** in $CHCl_3$.

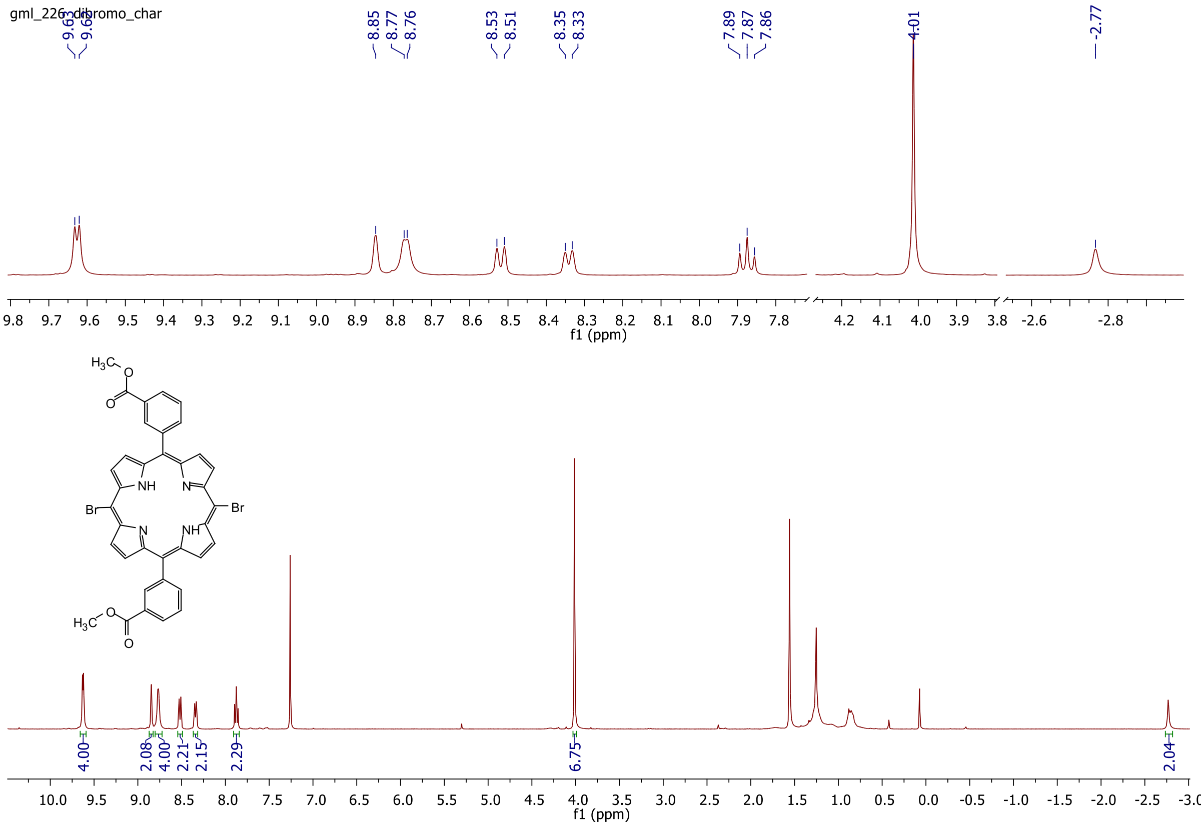

Figure S22: ^{1}H NMR of compound **17b** in $CDCl_3$.

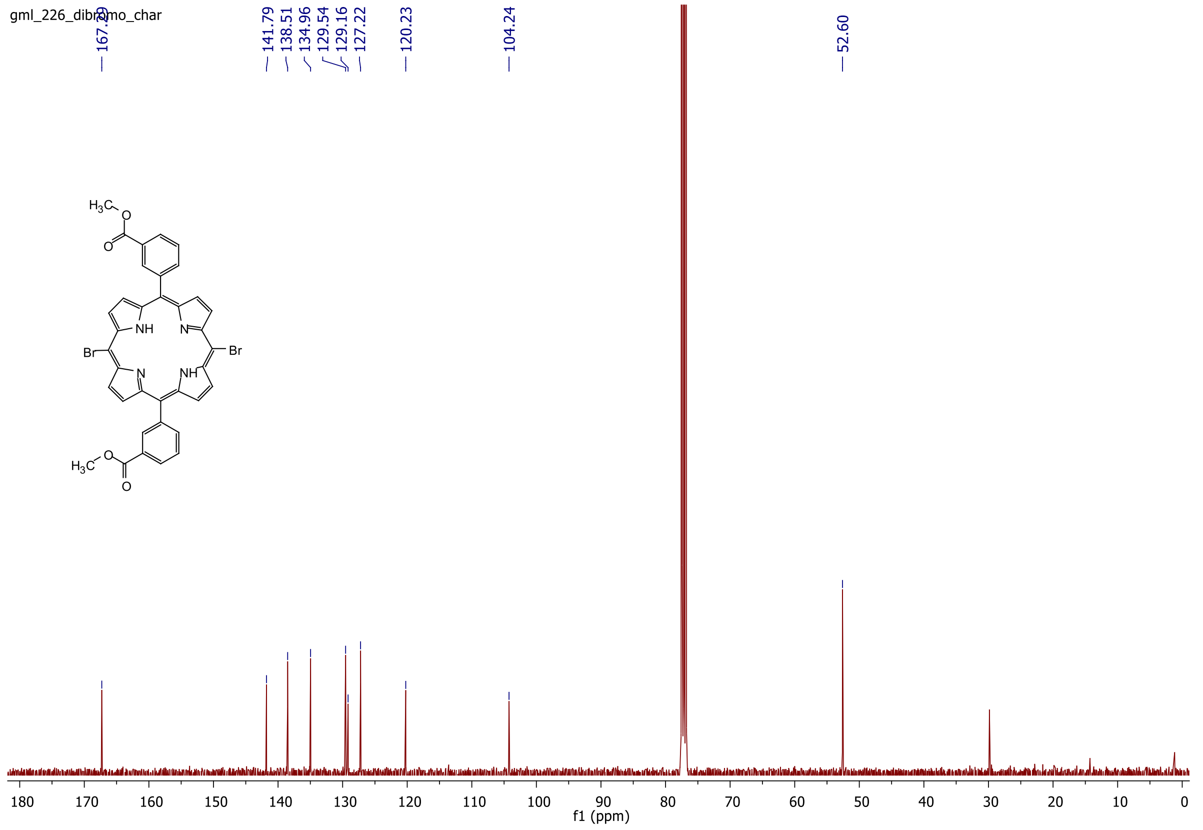

Figure S23: ^{13}C NMR of compound **17b** in $CDCl_3$.

Elemental Composition Report **Page 1**

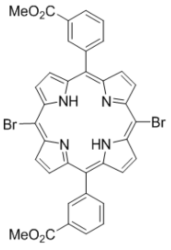

Single Mass Analysis
Tolerance = 50.0 PPM / DBE: min = -1.5, max = 200.0
Element prediction: Off
Number of isotope peaks used for i-FIT = 5

Monoisotopic Mass, Odd and Even Electron Ions
63 formula(e) evaluated with 1 results within limits (up to 10 best isotopic matches for each mass)
Elements Used:
C: 0-36 H: 0-25 N: 0-4 O: 0-4 Br: 0-2

Gemma Locke (MSe), GML-226-F1-dibromo
Q-TOF20180322MF012 38 (0.704) AM (Cen,8, 80.00, Ht,10000.0,1570.68,0.70); Sm (SG, 2x3.00); Sb (15,10.00); Cm (3:67-(47:57+38)) TOF MS LD+ 1.10e+003

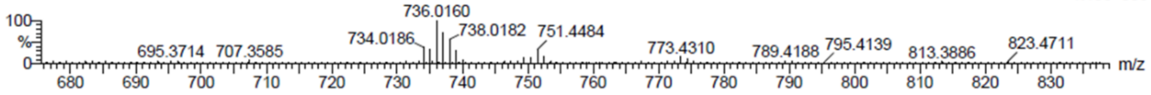

Minimum:				-1.5			
Maximum:		5.0	50.0	200.0			
Mass	Calc. Mass	mDa	PPM	DBE	i-FIT	i-FIT (Norm)	Formula
734.0186	734.0164	2.2	3.0	26.0	119.5	0.0	C36 H24 N4 O4 Br2

Figure S24: Mass spectrum of compound **17b** in $CHCl_3$.

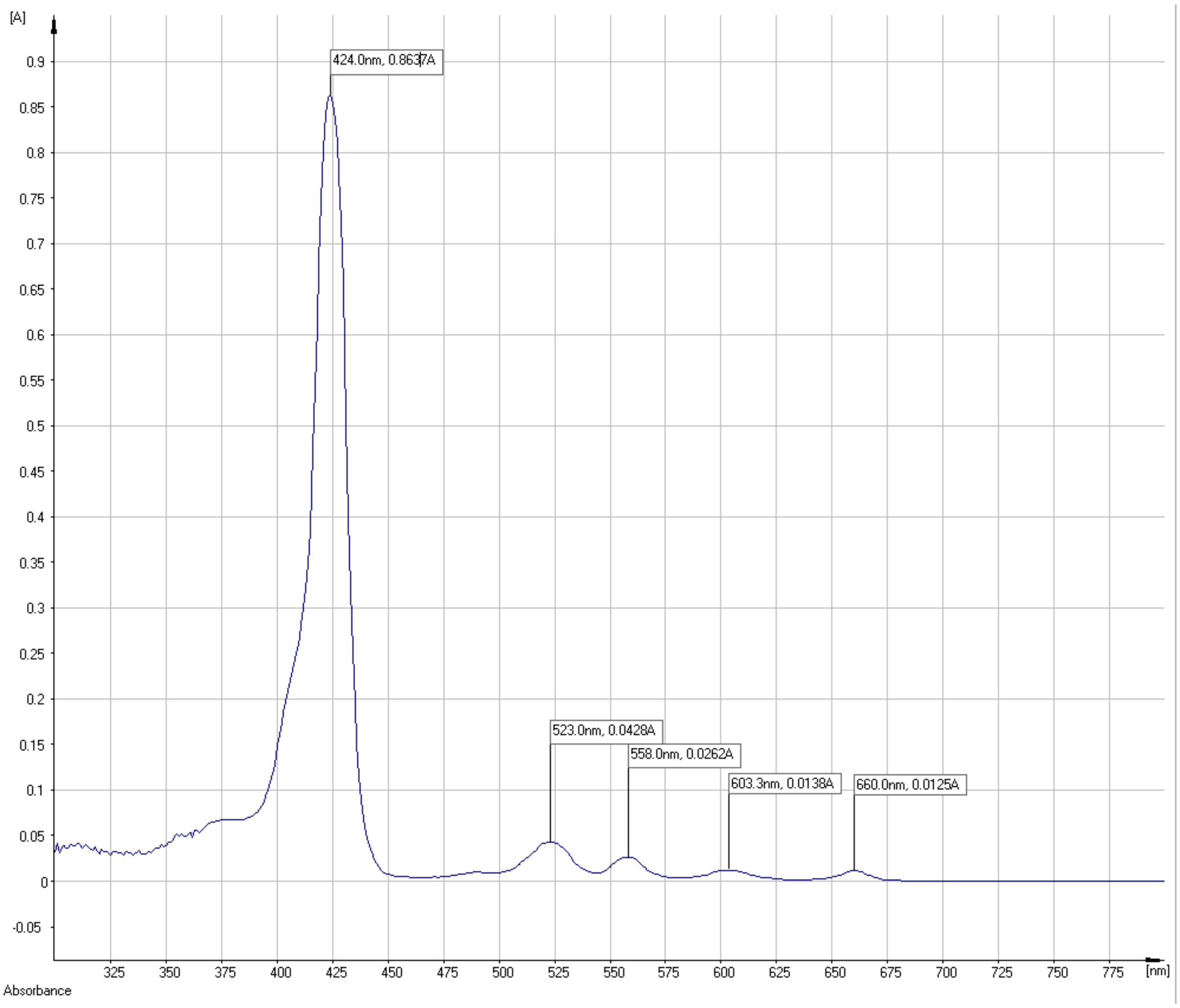

Figure S25: UV–vis spectrum of compound **17b** in $CHCl_3$.

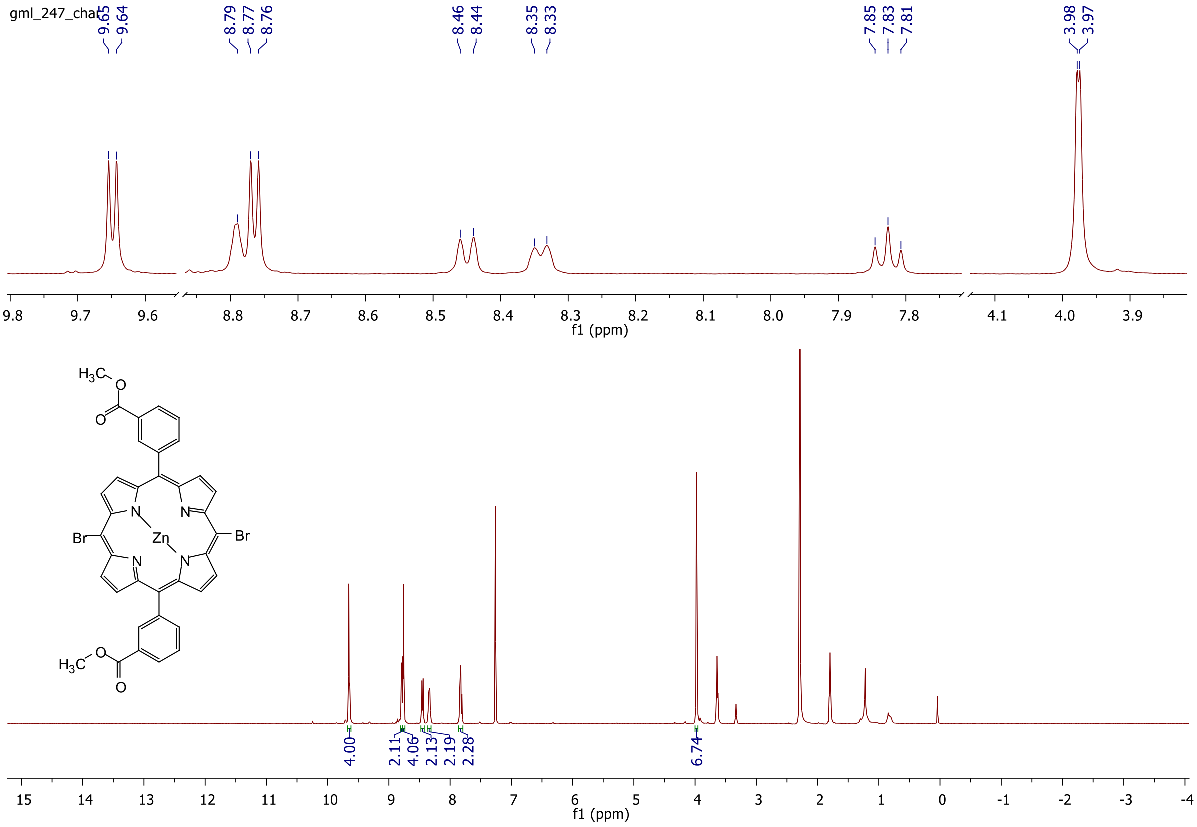

Figure S26: ^{1}H NMR of compound **17c** in $CDCl_3$.

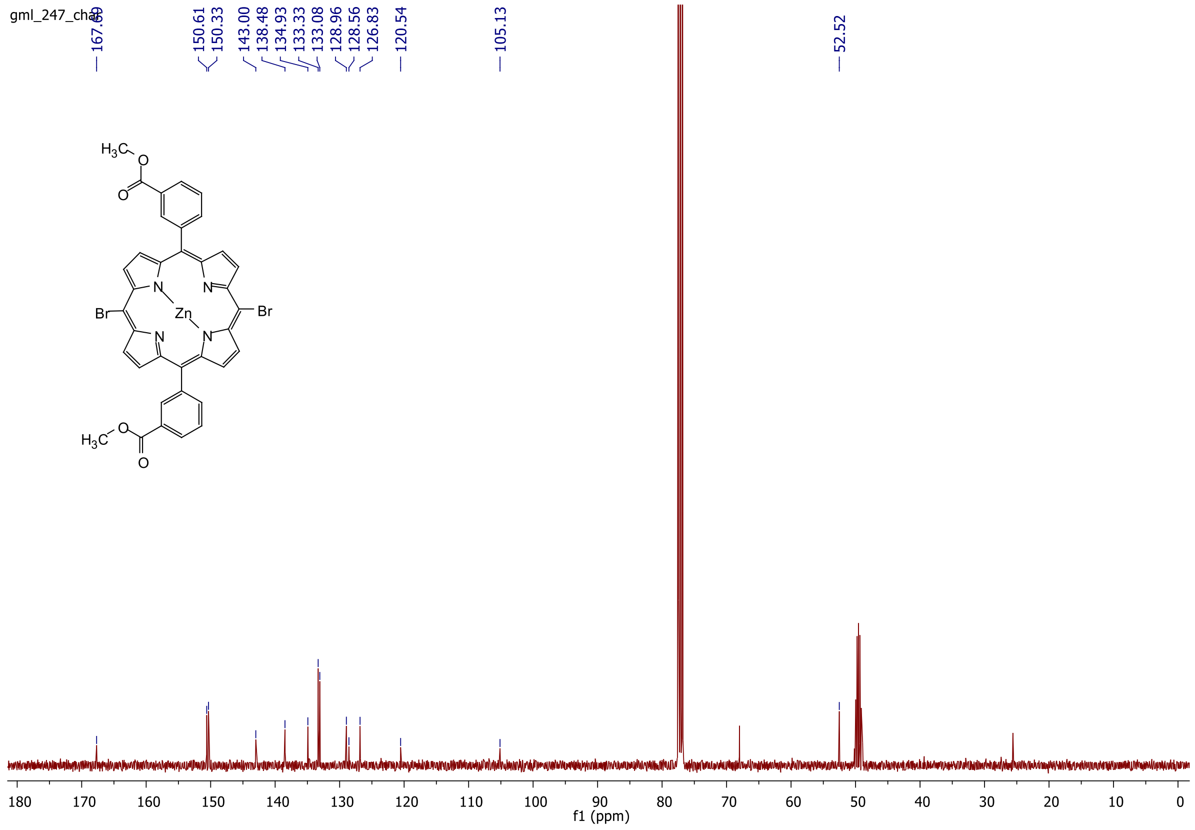

Figure S27: ^{13}C NMR of compound **17c** in $CDCl_3$.

Elemental Composition Report **Page 1**

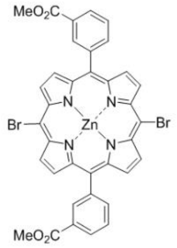

Single Mass Analysis
Tolerance = 50.0 PPM / DBE: min = -1.5, max = 200.0
Element prediction: Off
Number of isotope peaks used for i-FIT = 5

Monoisotopic Mass, Odd and Even Electron Ions
200 formula(e) evaluated with 1 results within limits (up to 10 best isotopic matches for each mass)
Elements Used:
C: 0-36 H: 0-22 N: 0-4 O: 0-4 Br: 0-2 Zn: 0-2

Gemma Locke (MSe), GML-247
Q-TOF20180322MF013 59 (1.093) AM (Cen,8, 80.00, Ht,10000.0,1570.68,0.70); Sm (SG, 2x3.00); Sb (15,10.00); Cm (7:112-(68:89+95)) TOF MS LD+ 6.25e+002

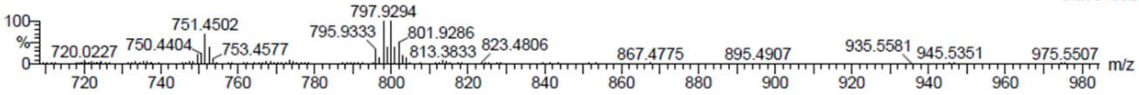

Minimum:				-1.5			
Maximum:		5.0	50.0	200.0			
Mass	Calc. Mass	mDa	PPM	DBE	i-FIT	i-FIT (Norm)	Formula
795.9333	795.9299	3.4	4.3	27.0	49.9	0.0	C36 H22 N4 O4 Br2 Zn

Figure S28: Mass spectrum of compound **17c** in $CHCl_3$.

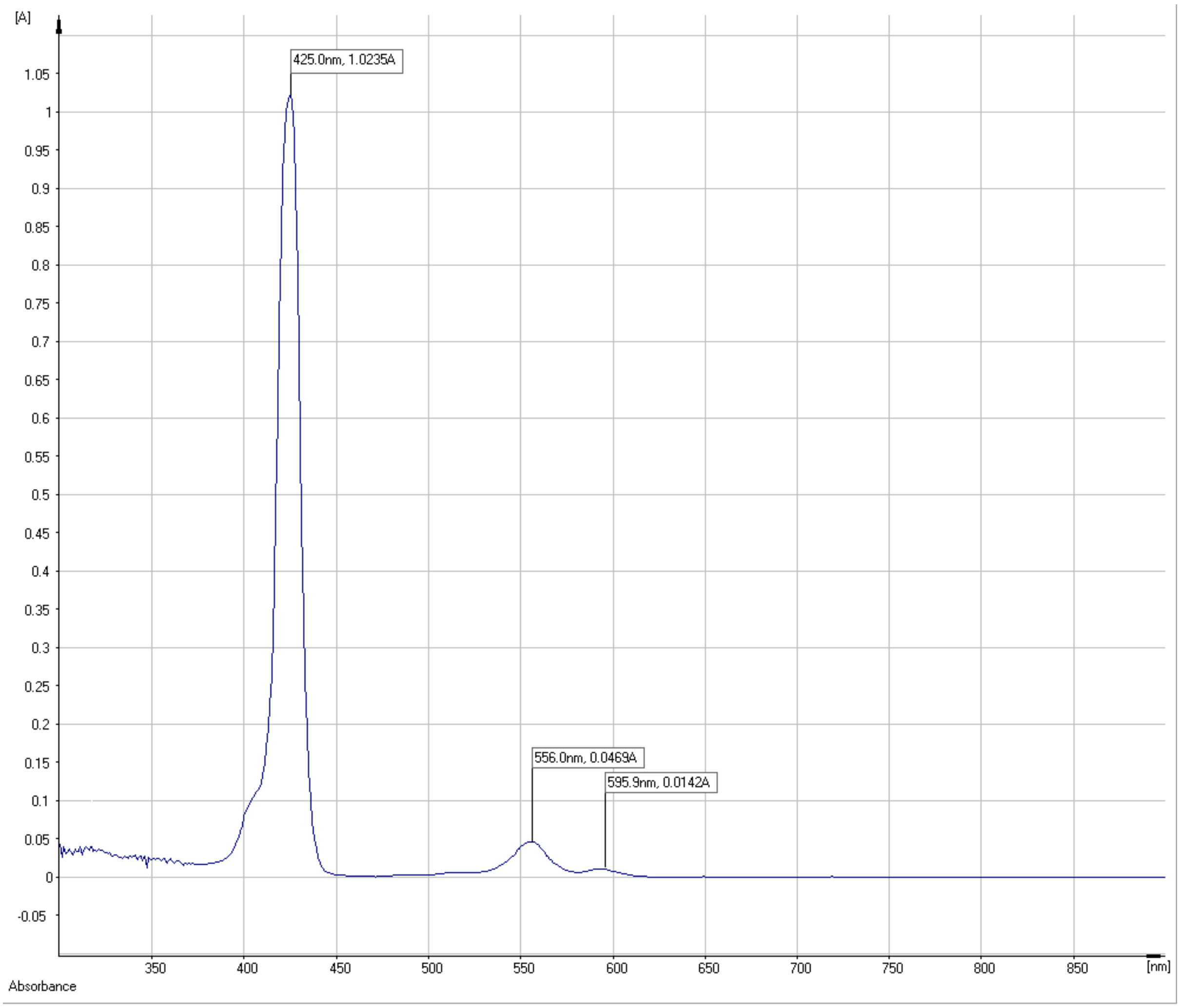

Figure S29: UV–vis spectrum of compound **17c** in $CHCl_3$.

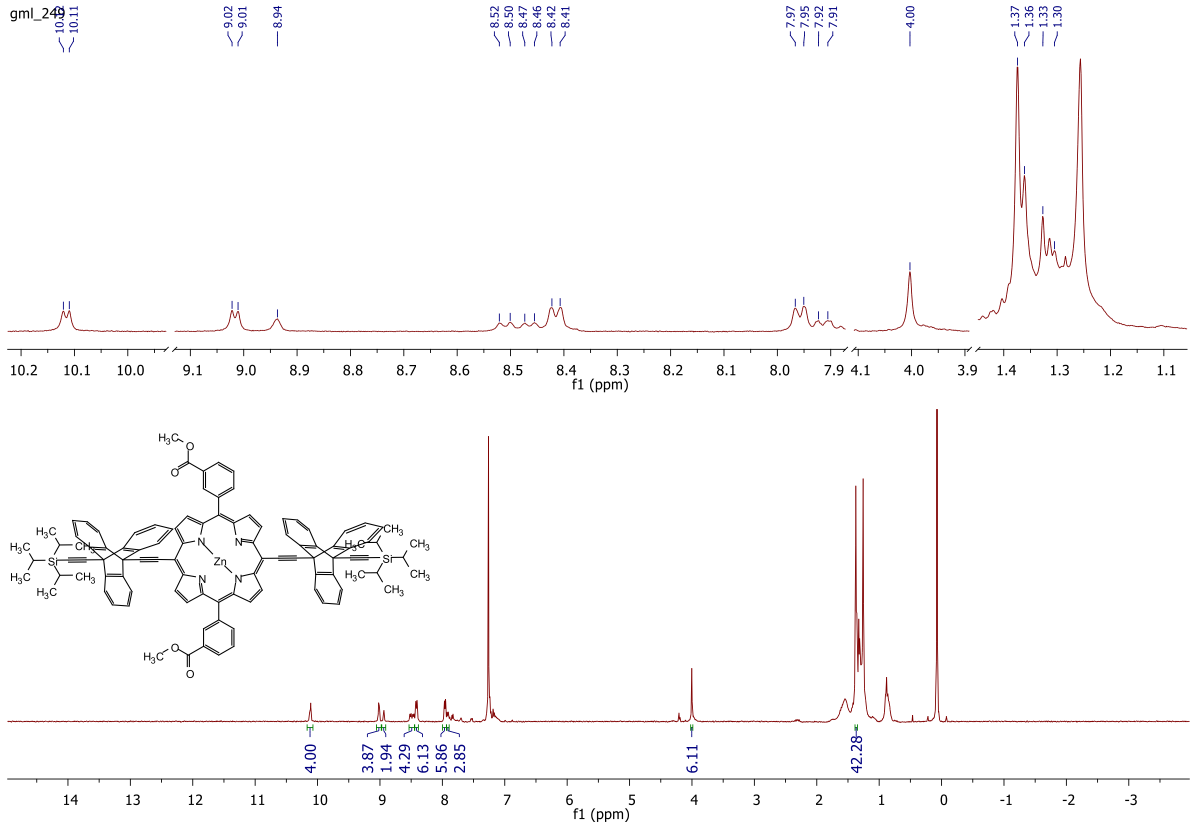

Figure S30: ^{1}H NMR of compound **18** in $CDCl_3$.

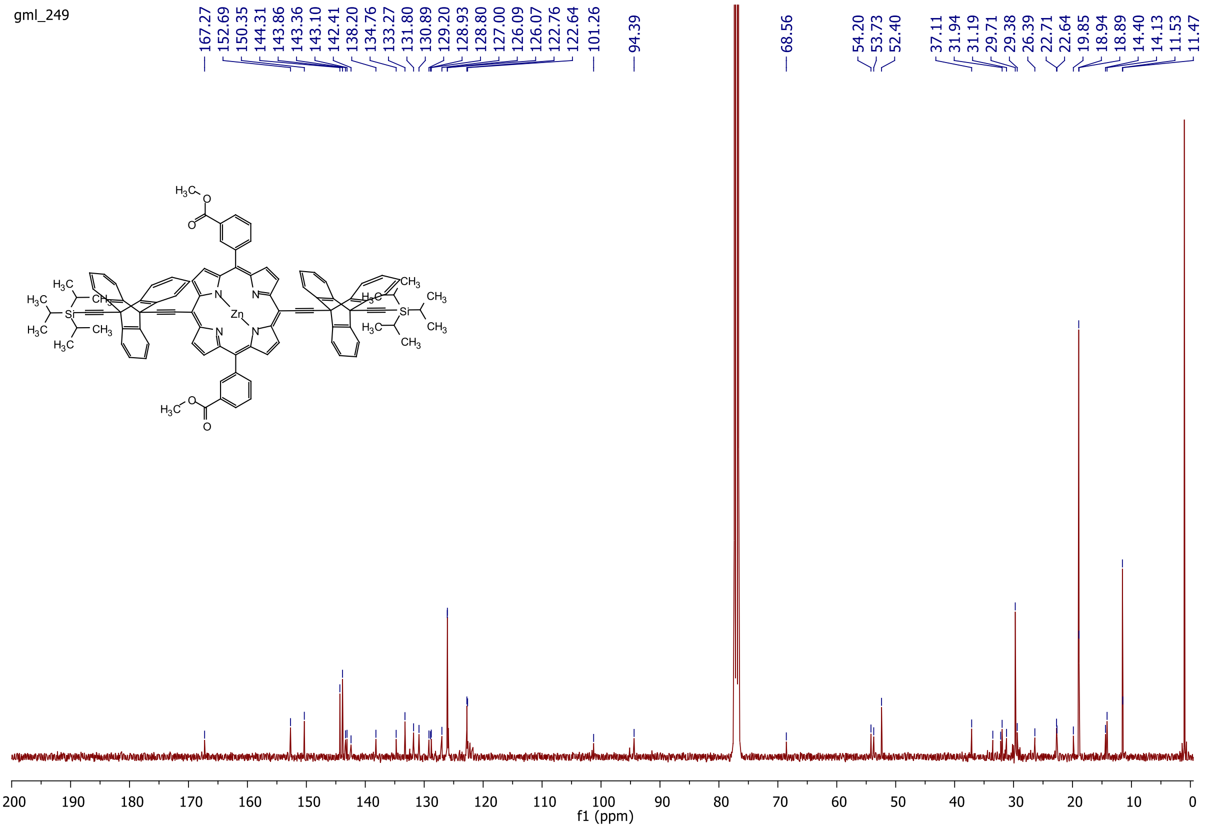

Figure S31: ^{13}C NMR of compound **18** in $CDCl_3$.

Elemental Composition Report **Page 1**

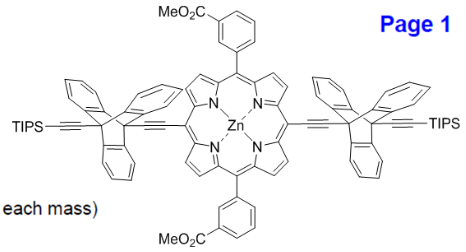

Single Mass Analysis
Tolerance = 10.0 PPM / DBE: min = -1.5, max = 200.0
Element prediction: Off
Number of isotope peaks used for i-FIT = 5

Monoisotopic Mass, Odd and Even Electron Ions
1697 formula(e) evaluated with 1 results within limits (up to 10 best isotopic matches for each mass)
Elements Used:
C: 0-102 H: 0-88 N: 0-4 O: 0-4 Si: 0-2 Zn: 0-2 Br: 0-2

Gemma Locke (MSe), GML-249-F2
Q-TOF20180325MF006 15 (0.278) AM (Cen,6, 80.00, Ht,10000.0,1570.68,0.70); Sm (SG, 2x3.00); Sb (15,10.00); Cm (1:51) TOF MS LD+ 3.56e+002

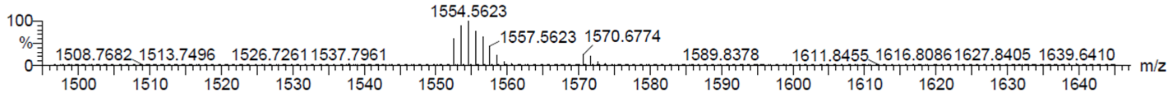

Minimum:				-1.5			
Maximum:		5.0	10.0	200.0			
Mass	Calc. Mass	mDa	PPM	DBE	i-FIT	i-FIT (Norm)	Formula
1552.5616	1552.5636	-2.0	-1.3	63.0	84.4	0.0	C102 H88 N4 O4 Si2 Zn

Figure S32: Mass spectrum of compound **18** in $CHCl_3$.

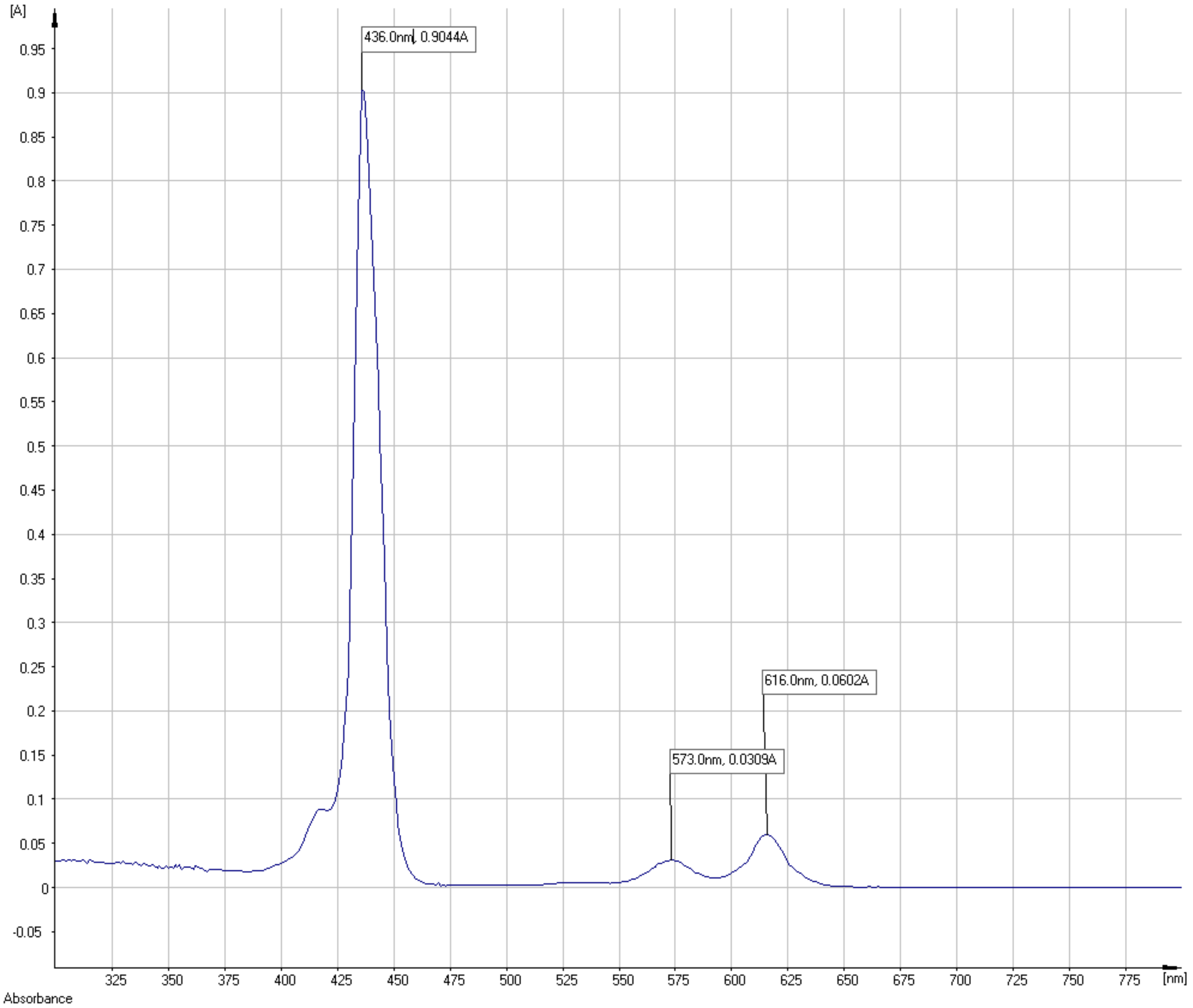

Figure S33: UV–vis spectrum of compound **18** in $CHCl_3$.

4. X-ray crystal structure determinations

Table S1: Details of XRD data refinement for compound **5** and **16**.

Compound	**5**	**16**
Empirical formula	$C_{18}H_{21}Si$	$C_{100}H_{90}N_8NiZn$
Formula weight	265.44	1527.87
Temperature/K	100(2)	100(2)
Crystal system	Triclinic	Triclinic
Space group	$P\bar{1}$	$P\bar{1}$
a/Å	10.9626(4)	10.5794(9)
b/Å	11.8640(4)	12.0811(10)
c/Å	12.8821(5)	31.213(3)
α/°	90.244(2)	90.007(2)
β/°	93.352(2)	94.017(2)
γ/°	98.157(2)	90.367(2)
Volume/Å^3	1655.53(10)	3979.4(6)
Z	4	2
$\rho_{calc}g/cm^3$	1.065	1.275
μ/mm^{-1}	1.112	0.593
F(000)	572.0	1608.0
Crystal size/mm^3	0.30×0.15×0.15	0.30×0.30×0.30
Radiation	CuKα	MoKα
Wavelength/Å	λ = 1.54178	λ = 0.71073
2θ/°	6.874–133.814	1.308–50.5
Reflections collected	36162	85838
Independent reflections	5788	14446
R_{int}	0.0696	0.0383
R_{sigma}	0.0442	0.0279
Restraints	6	40
Parameters	403	891
GooF	1.058	1.053

R_1 [I>=2σ (I)]	0.0808	0.0695
wR_2 [I>=2σ (I)]	0.2360	0.1631
R_1 [all data]	0.0972	0.0920
wR_2 [all data]	0.2531	0.1797
Largest peak/e Å^{-3}	0.94	0.98
Deepest hole/ e Å^{-3}	-0.45	-0.94

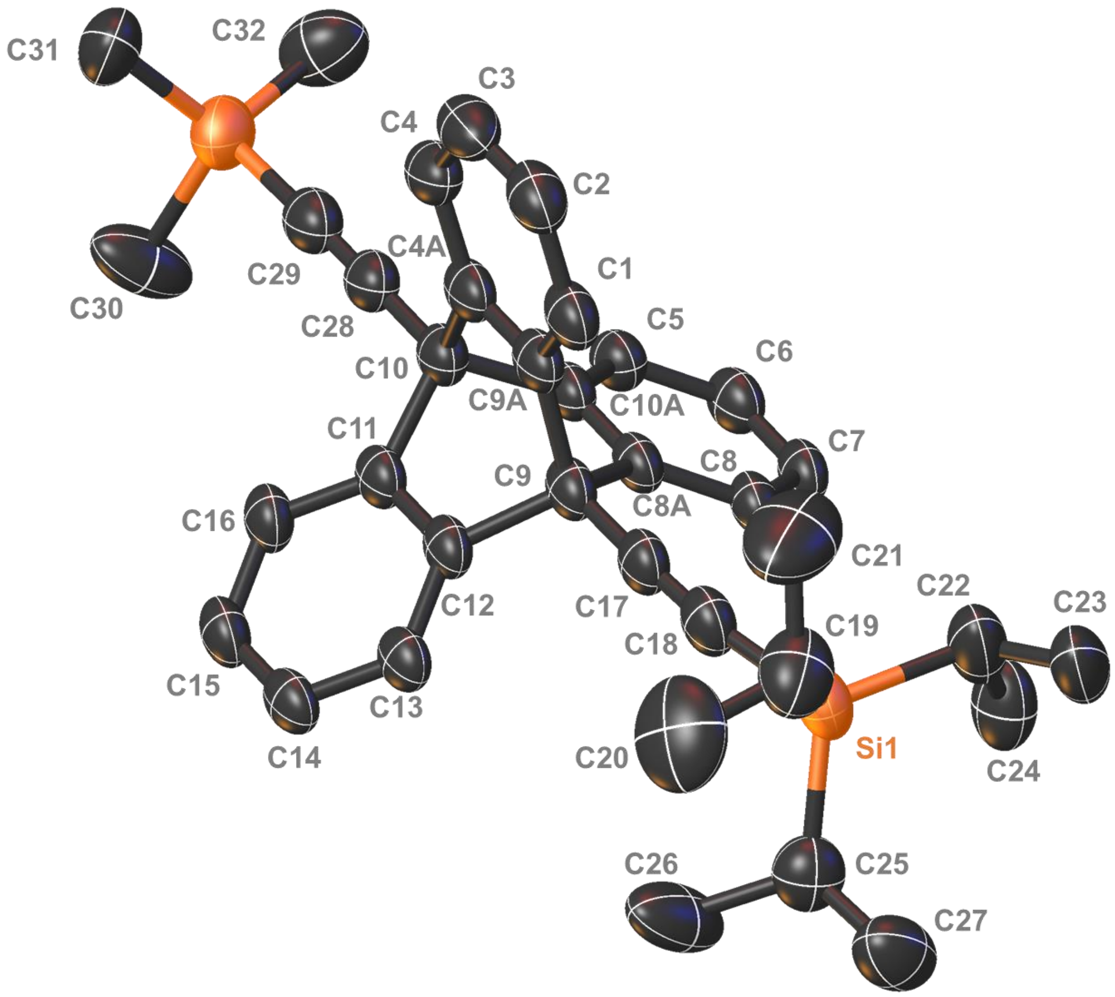

Figure S34: Labelled molecular view of **5**. Hydrogens atoms and minor disorder are omitted for clarity (thermal displacement 50%).

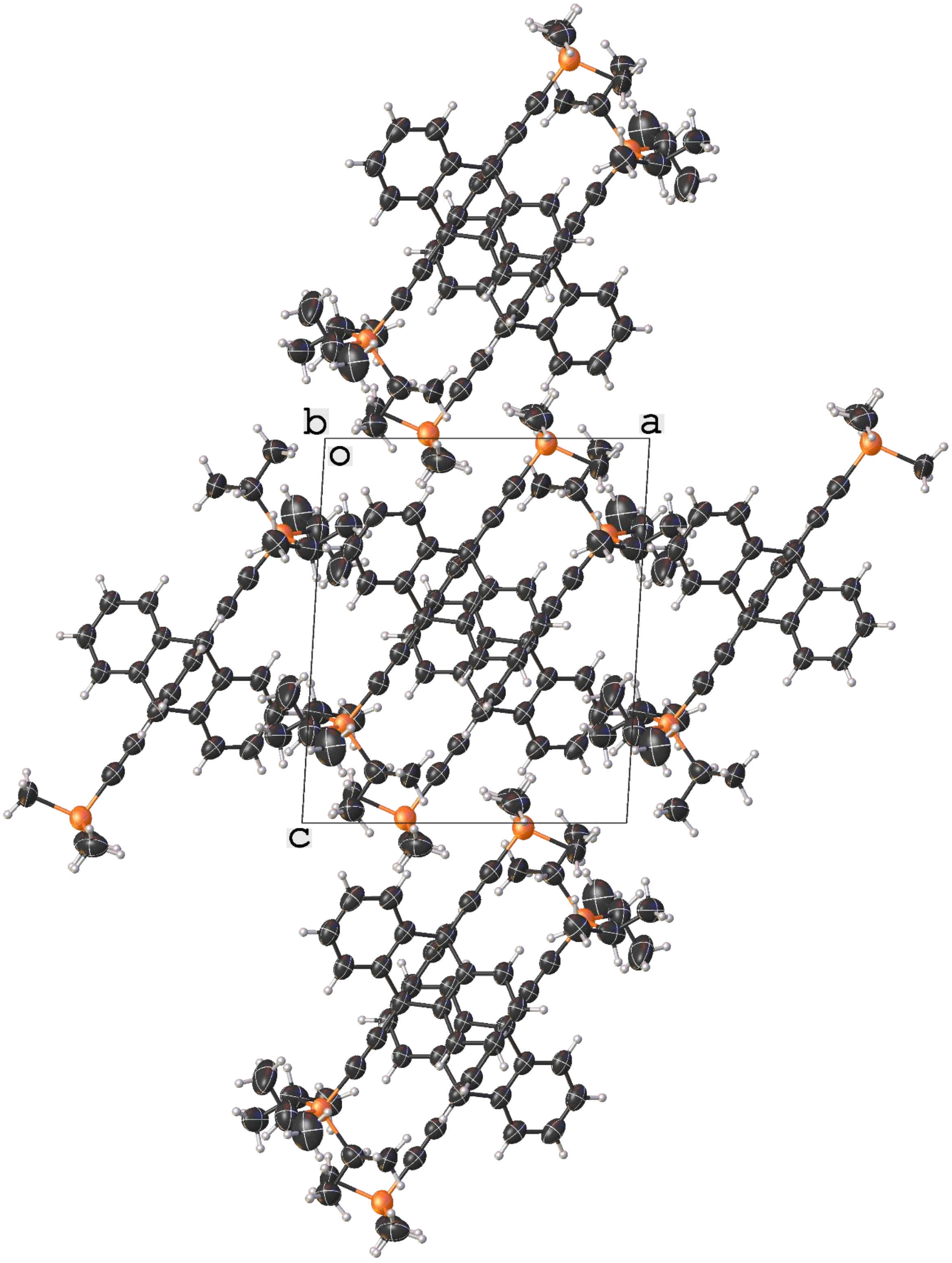

Figure S35: Crystal packing of **5** looking down the *b*-axis showing the stacking between the triptycene molecules.

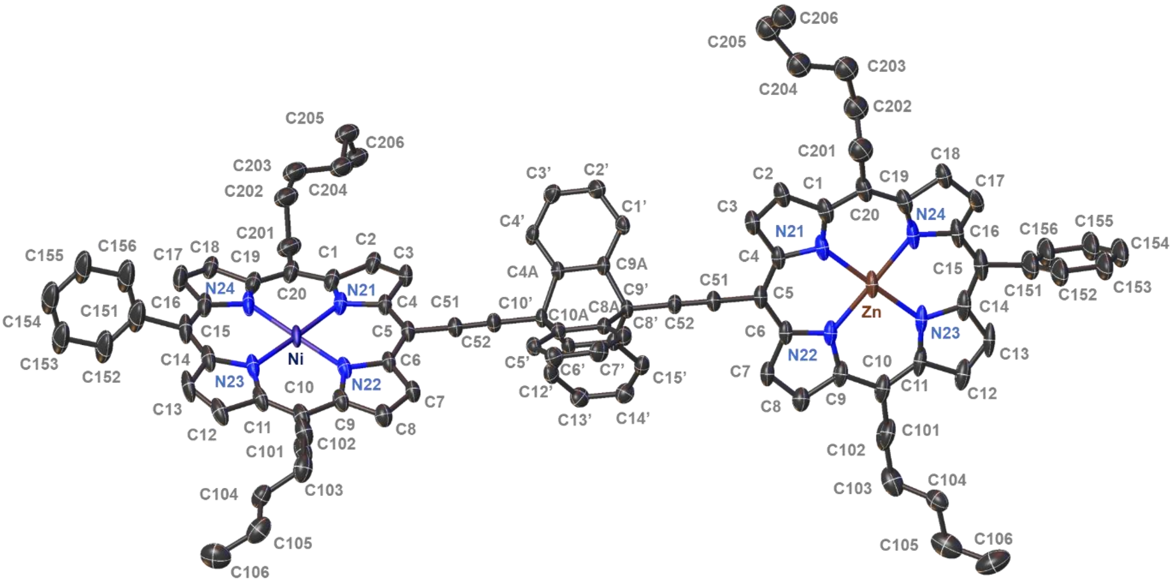

Figure S36: Labelled molecular view of **16**. Hydrogens atoms and minor disorder are omitted for clarity (thermal displacement 50%).

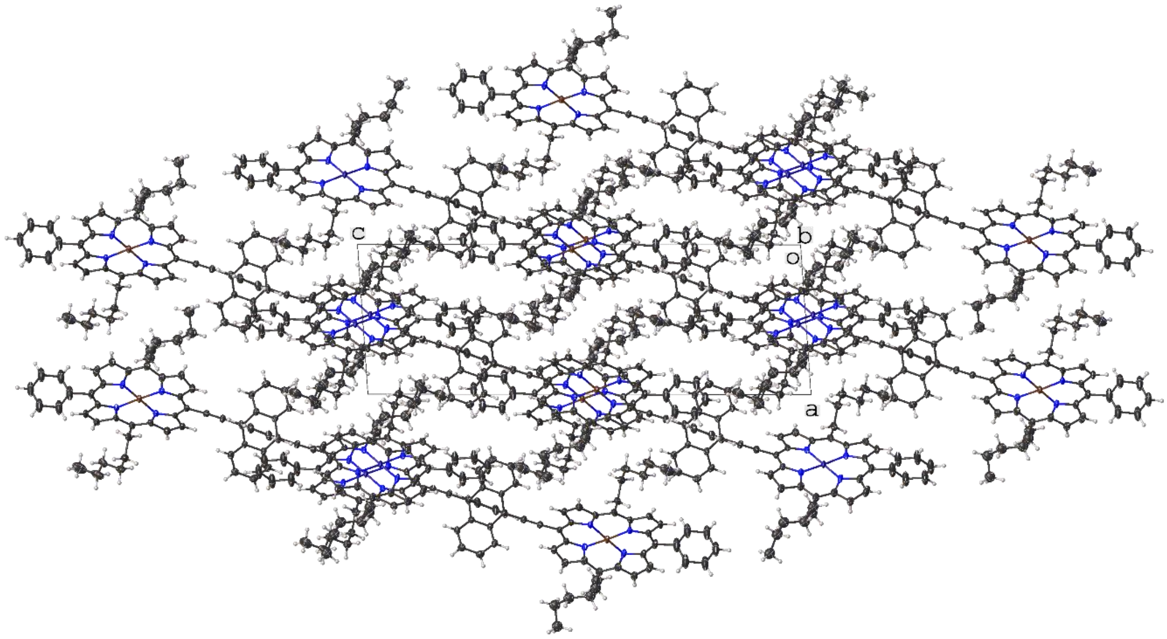

Figure S37: Crystal packing of **16** looking down the *b*-axis showing the stacking between the porphyrin moieties.

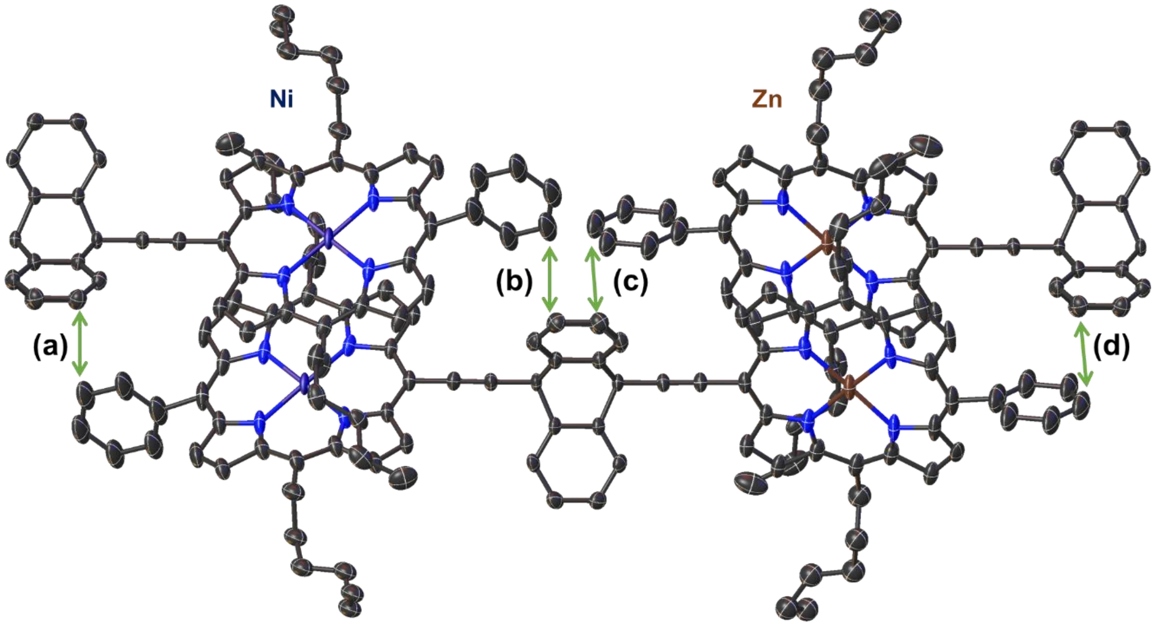

Figure S38: Overview of intermolecular interactions between triptycene aromatic rings and the phenyl rings of the porphyrin macrocycles in **16**. Hydrogens atoms and minor disorder are omitted for clarity (thermal displacement 50%).

5. Additional illustrations

The difference in the UV–vis and fluorescence spectra was also investigated for the porphyrin-triptycene-porphyrin dimer **7** and the triptycene-porphyrin-triptycene dimer **18**. The triptycene dimer **18** shows a slight redshift in the UV-Vis spectrum of 3, 9 and 6 nm for the three signals, respectively, potentially from the combined effect of the two ethynyl moieties and the electron withdrawing CO_2Me groups (Figure S39). The red shift was reversed for the emission spectrum, as a 5 nm hypochromic shift of the porphyrin dimer **7** can be seen with respect to the triptycene dimer **18** (Figure S40). A reduced intensity is also observed for the porphyrin dimer **7** potentially due to π-π interactions between the porphyrin units of two dimer molecules.

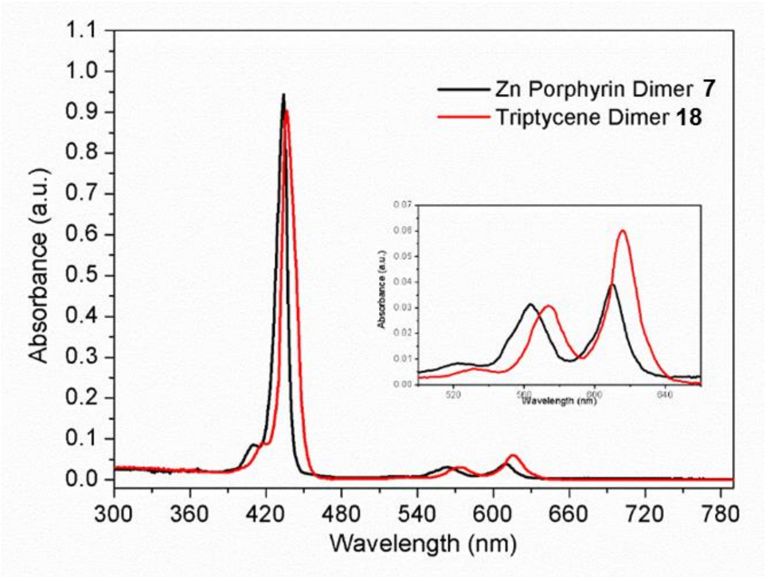

Figure S39: UV–vis of the zinc porphyrin dimer **7** *vs.* the triptycene dimer **18** in $CHCl_3$.

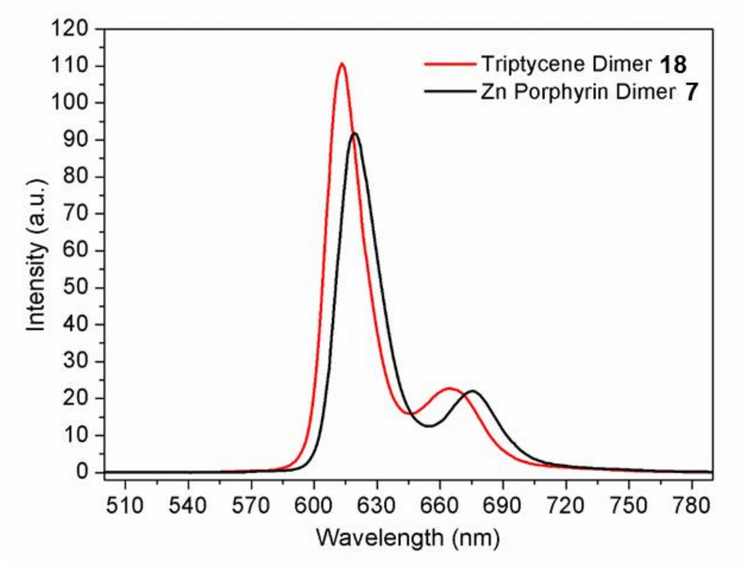

Figure S40: Emission spectra of the zinc porphyrin dimer **7** *vs.* the triptycene dimer **18** in $CHCl_3$.

6. References

1. Adcock, W.; Iyer, V. S. *J. Org. Chem.* **1988**, *53*, 5259–5266.
2. Miyaura, N.; Suzuki, A. *Chem. Rev.* **1995**, *95*, 2457–2483.
3. Sonogashira, K. *J. Organomet. Chem.* **2002**, *653*, 46−49.
4. Takita, M. Oi, R.; Kanazawa, J.; Muranaka, A.; Wang, C.; Uchiyama, M. *Chem. Sci.* **2019**, *10*, 6107–6112.
